# Evolution and expansion of the *Mycobacterium tuberculosis *PE and PPE multigene families and their association with the duplication of the ESAT-6 (*esx*) gene cluster regions

**DOI:** 10.1186/1471-2148-6-95

**Published:** 2006-11-15

**Authors:** Nicolaas C Gey van Pittius, Samantha L Sampson, Hyeyoung Lee, Yeun Kim, Paul D van Helden, Robin M Warren

**Affiliations:** 1 DST/NRF Centre of Excellence in Biomedical Tuberculosis Research, US/MRC Centre for Molecular and Cellular Biology, Division of Molecular Biology and Human Genetics, Department of Biomedical Sciences, Faculty of Health Sciences, Stellenbosch University, Tygerberg, South Africa; 2 Department of Molecular Microbiology and Infection, Centre for Molecular Microbiology and Infection, Imperial College London, South Kensington Campus, London, SW7 2AZ, UK; 3 Department of Biomedical Laboratory Science, College of Health Science, Yonsei University, Kangwon-do, Korea

## Abstract

**Background:**

The PE and PPE multigene families of *Mycobacterium tuberculosis *comprise about 10% of the coding potential of the genome. The function of the proteins encoded by these large gene families remains unknown, although they have been proposed to be involved in antigenic variation and disease pathogenesis. Interestingly, some members of the PE and PPE families are associated with the ESAT-6 (*esx*) gene cluster regions, which are regions of immunopathogenic importance, and encode a system dedicated to the secretion of members of the potent T-cell antigen ESAT-6 family. This study investigates the duplication characteristics of the PE and PPE gene families and their association with the ESAT-6 gene clusters, using a combination of phylogenetic analyses, DNA hybridization, and comparative genomics, in order to gain insight into their evolutionary history and distribution in the genus *Mycobacterium*.

**Results:**

The results showed that the expansion of the PE and PPE gene families is linked to the duplications of the ESAT-6 gene clusters, and that members situated in and associated with the clusters represent the most ancestral copies of the two gene families. Furthermore, the emergence of the repeat protein PGRS and MPTR subfamilies is a recent evolutionary event, occurring at defined branching points in the evolution of the genus *Mycobacterium*. These gene subfamilies are thus present in multiple copies only in the members of the *M. tuberculosis *complex and close relatives. The study provides a complete analysis of all the PE and PPE genes found in the sequenced genomes of members of the genus *Mycobacterium *such as *M. smegmatis*, *M. avium paratuberculosis*, *M. leprae*, *M. ulcerans*, and *M. tuberculosis*.

**Conclusion:**

This work provides insight into the evolutionary history for the PE and PPE gene families of the mycobacteria, linking the expansion of these families to the duplications of the ESAT-6 (*esx*) gene cluster regions, and showing that they are composed of subgroups with distinct evolutionary (and possibly functional) differences.

## Background

**Figure 1:**
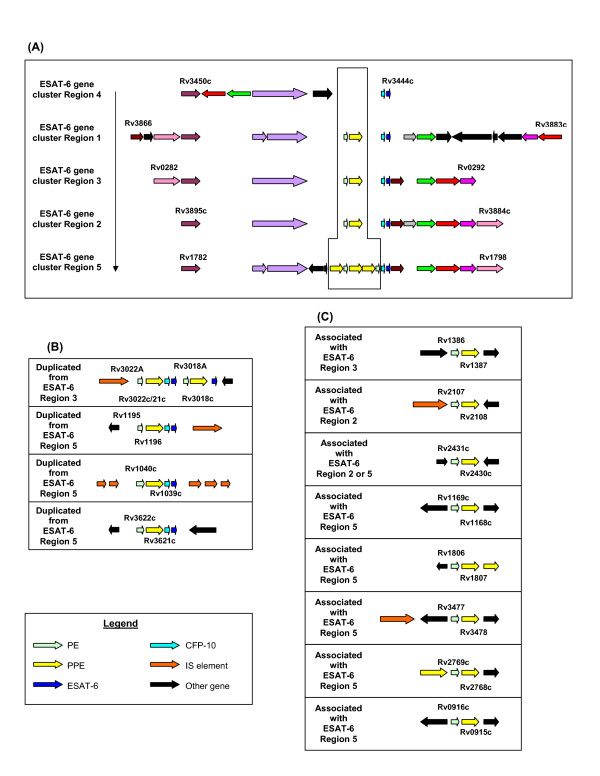
**Genomic organization of the PE and PPE genes associated with the *Mycobacterium tuberculosis *ESAT-6 (*esx*) gene clusters**. Open reading frames are represented by blocked arrows indicating direction of transcription, with the different colors reflecting specific gene families and the length of the arrow reflecting the relative lengths of the genes. (A) Schematic representation of the PE and PPE genes situated within the ESAT-6 (*esx*) gene cluster regions. The vertical arrow indicates the direction of duplication of the ESAT-6 (*esx*) gene cluster regions, from region 4, 1, 3, 2 and lastly 5 in descending order. The positions of the PE (small arrow in light green) and PPE (larger arrow in yellow) genes are blocked, (B) Schematic representation of the PE and PPE genes duplicated from the ESAT-6 (*esx*) gene cluster regions, with the positions of the ESAT-6 and CFP-10 genes indicated, (C) Schematic representation of the PE and PPE genes associated with the ESAT-6 (*esx*) gene cluster regions (''associated with'' denotes genes which are hypothesized to have been duplicated from ESAT-6 (esx) gene cluster regions, as they are very homologous to their paralogues within the ESAT-6 (*esx*) gene clusters and have the same paired genomic orientation – see also Table 2).

The genome of *Mycobacterium tuberculosis *contains five copies of the immunopathologically-important ESAT-6 (*esx*) gene clusters [1]. Each gene cluster encodes proteins involved in energy provision for active transport, membrane pore formation and protease processing, which assembles to form a dedicated biosynthesis, transport and processing system for the secretion of the potent T-cell antigens belonging to the ESAT-6 protein family [1-9]. Although other, chromosomally unlinked, but homologous, genes seem to play a role in this novel secretory system [10,11], there are two families of genes present within the clusters which have no apparent function in the secretion system, namely the PE and PPE gene families (Figure 1A).

**Figure 2:**
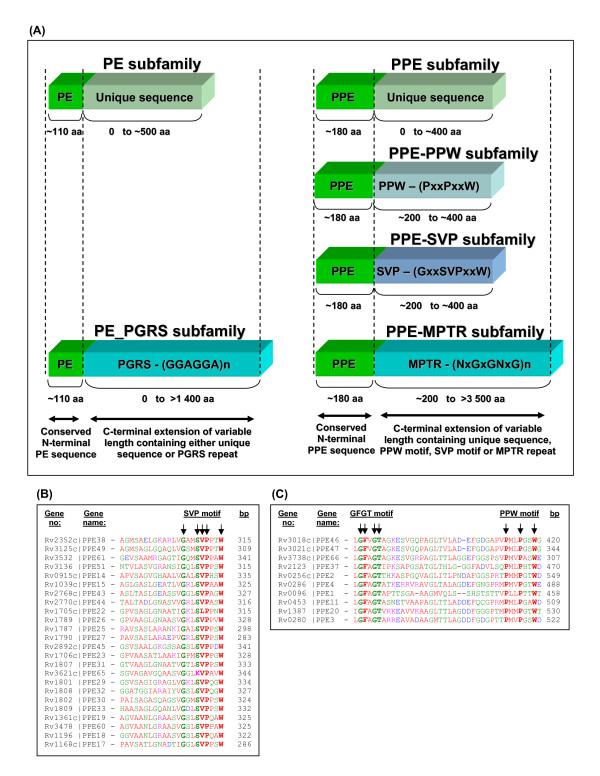
**PE/PPE gene structure**. (A) Diagrammatic representation of the gene structure of the members of the PE and PPE gene family, showing conserved N-terminal domains, motif positions and differences between different subfamilies found in the two families [12,16]. (B) Alignment of the region surrounding the SVP motif Gly-X-X-Ser-Val-Pro-X-X-Trp in the members of the PPE-SVP subfamily. (C) Alignment of the region surrounding the GFGT motif (Gly-Phe-X-Gly-Thr) and PPW motif (Pro-X-X-Pro-X-X-Trp) in the members of the PPE-PPW subfamily.

The PE and PPE gene families of *M. tuberculosis *encode large multi-protein families (99 and 69 members, respectively) of unknown function [12,13]. These protein families comprise about 10% of the coding potential of the genome of *M. tuberculosis *[12]. The PE family is characterized by the presence of a proline-glutamic acid (PE) motif at positions 8 and 9 in a very conserved N-terminal domain of approximately 110 amino acids [14]. Similarly, the PPE family also contains a highly conserved, but unique, N-terminal domain of approximately 180 amino acids, with a proline-proline-glutamic acid (PPE) motif at positions 7–9 (Figure 2A) [12]. Although the N-terminal domains are conserved within each family, there is very little N-terminal homology between the two different families. The C-terminal domains of both of these protein families are of variable size and sequence and frequently contain repeat sequences of different copy numbers [14].

Both the PE and PPE protein families can be divided into subfamilies according to the homology and presence of characteristic motifs in their C-terminal domains [14]. The polymorphic GC-rich-repetitive sequence (PGRS) [15] subfamily of the PE family is the largest subfamily (65 members) and contains proteins with multiple tandem repeats of a glycine-glycine-alanine (Gly-Gly-Ala) or a glycine-glycine-asparagine (Gly-Gly-Asn) motif in the C-terminal domain [14]. The other PE subfamily (34 members) consists of proteins with C-terminal domains of low homology [14]. The PPE family can be broadly divided into four subfamilies [14,16] of which the PPE-SVP subfamily is the largest (24 members). The proteins of this subfamily are characterized by the motif Gly-X-X-Ser-Val-Pro-X-X-Trp between position 300 and 350 in the amino acid sequence (Figure 2B). The major polymorphic tandem repeat (MPTR) PPE subfamily is the second largest (23 members) and contains multiple C-terminal repeats of the motif Asn-X-Gly-X-Gly-Asn-X-Gly, encoded by a consensus repeat sequence GCCGGTGTTG, separated by 5 bp spacers [17,18]. The third subfamily (10 members), recently identified by Adindla and Guruprasad [16], is characterized by a conserved 44 amino acid residue region in the C-terminus comprising of highly conserved Gly-Phe-X-Gly-Thr and Pro-X-X-Pro-X-X-Trp sequence motifs (Figure 2C, named the "PPE-PPW" subfamily for the purpose of this study). The last PPE subfamily (12 members) consists of proteins with a low percentage of homology at the C-terminus [14].

An early paper by Doran and coworkers [19] suggested that the members of the PPE-MPTR family were likely to be cell wall associated. Association of a PPE protein with the mycobacterial cell wall was first demonstrated experimentally for the PPE-MPTR protein Rv1917c (PPE34), which was also demonstrated to be at least partly exposed on the cell surface [20]. It has subsequently been shown that certain PE_PGRS proteins are cell-surface constituents [21-23] which influence the cellular architecture and colony morphology [23] as well as the interactions of the organism with other cells [21]. More recently, it has been demonstrated that the PPE proteins Rv2108 (PPE36) and Rv3873 (PPE68) are also both cell-wall associated [24,25]. Furthermore, Pajon and coworkers [26] have identified at least one outer membrane anchoring domain with the potential to form a beta-barrel outer-membrane protein-like structure in 40 different PE and PPE proteins. It has yet to be shown whether all PE and PPE proteins localize to the cell wall, and secretion into the extracellular environment has not been ruled out.

Although the function of the 168 members of the PE and PPE protein families has not been established, various hypotheses have been advanced. The fact that these genes encode about 4% of the total protein species in the organism (if all genes are expressed), suggests that they most probably fulfill an important function or functions in the organism. The most widely-supported theory suggests the involvement of these proteins in antigenic variation due to the highly polymorphic nature of their C-terminal domains [12,14,27]. In agreement with this, sequence variation has been observed between the orthologues of the PE and PPE protein families in *in silico *analyses of the sequenced genomes of *M. tuberculosis *H37Rv, *M. tuberculosis *CDC1551 and *M. bovis *[28-30]. Extensive variation of a subset of PPE genes in clinical isolates of *M. tuberculosis *has also been observed (S. Sampson, unpublished results) and a recent study by Talarico *et al*. [31] found sequence variation for PE_PGRS33 (Rv1818c) in 68% of clinical isolates spanning all three *M. tuberculosis *principal genetic groups [32]. Additionally, Srivastava *et al*. [33] showed in an analysis of more than 300 clinical isolates of *M. tuberculosis *that the MPTR domain of the PPE gene Rv0355c (PPE8) displayed several polymorphisms. There is also ample evidence available for differential expression of members of the PE/PE_PGRS family between different strains of *M. tuberculosis *[34] as well as under different environmental and experimental conditions. [35-38]. However, the observed sequence variation and differential expression has yet to be related to antigenic variation.

An alternative way in which the PE and PPE proteins may interact with the host immune system is by the inhibition of antigen processing [12]. Some support for this hypothesis is provided by a report that a DNA vaccine construct based on the conserved N-terminal PE region of the PE_PGRS protein Rv1818c (PE_PGRS33) is able to elicit a cellular immune response, whereas a construct containing the whole PE_PGRS region is unable to do so [39], suggesting that the PGRS repeats are in some way able to influence antigen processing and presentation. This is supported by a recent follow-up study, in which Dheenadhayalan and coworkers [40] demonstrated that expression of the complete PE_PGRS33 protein in the non-pathogenic fast-growing *M. smegmatis*, causes the strain to survive better in infected macrophage cultures and mice than a parental strain or a strain expressing only the PE domain of the protein. Work done by Delogu *et al*. [23] proved that the PE domain of PE_PGRS33 is necessary for subcellular localization, while the PGRS domain, but not PE, affects the bacterial shape and colony morphology. It was also shown previously that an *M. bovis *BCG strain containing a transposon insertion in PE_PGRS33 could not infect (and survive in) macrophages and showed dispersed growth in liquid media [21]. Complementation of this mutant restored infectivity of macrophages as well as aggregative growth (clumping) in liquid media [21].

Other diverse clues to the potential functions of the members of these families exist. For example, Rodriguez and colleagues [41,42] have found that the PPE gene Rv2123 (PPE37) is upregulated under low iron conditions, leading to the hypothesis that this gene may encode a siderophore involved in iron uptake. One member of the PE_PGRS family, Rv1759c (wag22), has been characterized as a fibronectin binding protein [43,44]. Interestingly, the orthologue of this gene in the closely-related genome of *M. bovis *is a pseudogene, the absence of which could potentially play a role in influencing host or tissue tropism [30]. It was also shown that two *M. marinum *orthologues of the PE_PGRS subfamily are essential for replication in macrophages as well as persistence in granulomas [45]. More recently, an *M. avium *PPE protein (Rv1787/PPE25 orthologue), expressed only in macrophages, has been shown to influence macrophage vacuole acidification, phagosome-lysosome fusion and replication in macrophages; and to be associated with virulence in mice [36]. Additional data supports the notion that members of the PPE gene family may be involved in disease pathogenesis, as a transposon mutant of the PPE gene Rv3018c (PPE46) was attenuated for growth in macrophages [46]. Sassetti *et al*. [47], confirmed the importance of Rv3018c and identified a further 5 PPE genes (Rv0286/PPE4, Rv0755c/PPE12, Rv1753c/PPE24, Rv3135/PPE50 and Rv3343c/PPE54) and 3 PE genes (Rv0285/PE5, Rv0335c/PE6 and Rv1169c/PE11) as essential for *in vitro *growth in a transposon-mutagenesis-based screen, although a follow-up study by the same group [48] showed that only two PPE's (Rv1807/PPE31 and Rv3873/PPE68) and one PE (Rv3872/PE35) are specifically required for mycobacterial growth *in vivo *during infection of mice. The authors speculated that the fact that such a small fraction were detected in their system suggests either that most of these genes are able to functionally complement each other, or that they are required under conditions that were not tested. Interestingly, Rv3872 (PE35) and Rv3873 (PPE68), required for *in vivo *growth, are both situated within the ESAT-6 gene cluster region 1 [1], which has been previously shown to be involved in pathogenicity of the organism [4,6,8,49-51], while Rv0285 (PE5) and Rv0286 (PPE4), required for *in vitro *growth, are both situated within the ESAT-6 gene cluster region 3 [1]. Recently, Jain and coworkers [52] identified three PE_PGRS genes (Rv0977/PE_PGRS16, Rv0978c/PE_PGRS17 and Rv0980c/PE_PGRS18) and two PPE genes (Rv1801/PPE29 and Rv3021c/PPE47) to be up-regulated by at least 8-fold in human brain microvascular endothelial-cell-associated *M. tuberculosis *and showed that at least Rv0980c and Rv1801 are potentially required for endothelial-cell invasion and/or intracellular survival. This confirmed data by Talaat *at al*. [53] which identified the same PE_PGRS genes Rv0977, Rv0978c and Rv0980c to form part of a so-called *in vivo*-expressed genomic island that was highly expressed only *in vivo *and not *in vitro*.

The evolution and distribution of the members of the PE and PPE gene families in the genus *Mycobacterium*, as well as their association with the ESAT-6 gene cluster regions within these organisms are unknown. The only attempt to obtain some insight into the relationships among members of specifically the large PE_PGRS gene family was done in an analysis by Espitia *et al*. [44], in order to identify the closest relatives of a PE_PGRS sequence involved in fibronectin-binding. This resulted in an uninformative unrooted tree only suggesting a complex evolutionary history for this gene family.

Sequencing of the complete genomes of organisms has provided a wealth of information concerning phenotype and evolution. The information obtained from these sequencing projects can be used to trace the evolution of genes and gene families using comparative genomics. This study investigates the evolutionary history of the mycobacterial PE and PPE gene families using *in silico *sequence analyses, phylogenetic analyses, DNA hybridization and comparative genomics of a selected set of mycobacterial genome sequences. We attempt to answer the question of why and how these PE and PPE genes were duplicated, as well as provide insight into the relationship between these genes and the ESAT-6 (*esx*) gene clusters. We envisage that this data will provide a better understanding of the factors involved in the considerable expansion of the PE and PPE families, their evolutionary and functional relationship to the ESAT-6 (*esx*) gene cluster regions, and the evolution of the mycobacterial genome.

## Results and Discussion

### Identification of the most ancestral PE and PPE genes

#### The PE and PPE gene families are not present outside the genus *Mycobacterium*

In order to be able to construct a robust evolutionary history of the PE and PPE gene families through phylogenetic analysis, it is of critical importance to first identify the most ancestral representatives of both these families. These ancestral genes are used as the root for the construction of the relationship tree, and represents the origin of the family. Comparative genomics, during which the genomes of different species are compared to look for differences and similarities, is the tool of choice for the identification of orthologues of genes in these species. To date, 31 mycobacterial genome sequencing projects are in various stages of completion (see Table 1), representing a valuable resource for comparative genomics analyses within the genus *Mycobacterium*. A detailed examination of the sequenced genomes of species belonging to closely-related genera to the mycobacteria (e.g. *Corynebacteria*, *Nocardia *etc.) have shown that the PE and PPE genes are not found outside of the genus *Mycobacterium *(data not shown). This is in agreement with the published genome analyses of these organisms [54-60]. Where repetitive proteins with some homology to the PE and PPE gene families have been identified previously (e.g. nfa8180 in *Nocardia farcinica *and SAV5103, SAV6636, SAV6731, SAV7299 in *Streptomyces avermitilis *– see Ishikawa *et al*. [59]), this is merely due to unspecific alignment of the repetitive regions and these proteins do not contain the conserved N-terminal PE and PPE domains or the conserved PE and PPE motifs. The answer to the evolution and expansion of these multigene PE and PPE families thus lies within the genus *Mycobacterium*.

**Table 1:** Mycobacterial genome sequencing projects

**Organism**	**Size**	**%GC**	**Assigned genes**	**Website**
*Mycobacterium tuberculosis *H37Rv	4 411 532 bp	65.6	3993	
*Mycobacterium tuberculosis *CDC1551	4 403 837 bp	65.6	4246	
*Mycobacterium tuberculosis *C	4 276 000 bp	-	4039	
*Mycobacterium tuberculosis *F11	4 413 077 bp	65.6	3911	
*Mycobacterium tuberculosis *210	± 4 447 000 bp	-	-	
*Mycobacterium tuberculosis *K	-	-	-	
*Mycobacterium tuberculosis *Haarlem	-	-	-	
*Mycobacterium tuberculosis *Peruvian1	-	-	-	
*Mycobacterium tuberculosis *Peruvian2	-	-	-	
*Mycobacterium tuberculosis *W-148	-	-	-	
*Mycobacterium tuberculosis *A1	-	-	-	
*Mycobacterium tuberculosis *Ekat-4	-	-	-	
*Mycobacterium bovis *AF2122/97	4 345 492 bp	65.6	3953	
*Mycobacterium bovis *BCG Pasteur 1173P2	4 375 192 bp	65.6	-	
*Mycobacterium microti *OV254	± 4 400 000 bp	~64	-	
*M. canettii*	± 4 400 000 bp	~64	-	
*M. africanum*	± 4 400 000 bp	~64	-	
*Mycobacterium marinum *M	6 636 827 bp	65.7	-	
*Mycobacterium ulcerans *Agy99	5 631 606 bp	65.7	4281	
*Mycobacterium ulcerans*	± 4 600 000 bp	~65	-	
*M. leprae *TN	3 268 203 bp	57.8	1614	
*Mycobacterium avium *104	± 4 700 000 bp	69	-	
*Mycobacterium avium paratuberculosis *K-10	4 829 781 bp	69.2	4350	
*Mycobacterium smegmatis *mc2155	6 988 209 bp	67.4	6776	
*Mycobacterium flavenscens *Pyr-GCK	± 5 939 000 bp	67	5606	
*Mycobacterium vanbaalenii *Pyr-1	± 6 460 000 bp	68	6012	
*Mycobacterium sp *MCS	5 705 450 bp 215 077 bp (plasmid)	68	5615	
*Mycobacterium sp *KMS	± 6 228 000 bp	68	5891	
*Mycobacterium sp *JLS	± 6 040 000 bp	68	5711	
*M. abscessus *CIP 104536T	-	-	-	
*M. chelonae *CIP 104535	-	-	-	

#### Generation of a mycobacterial phylogenetic tree

**Figure 3:**
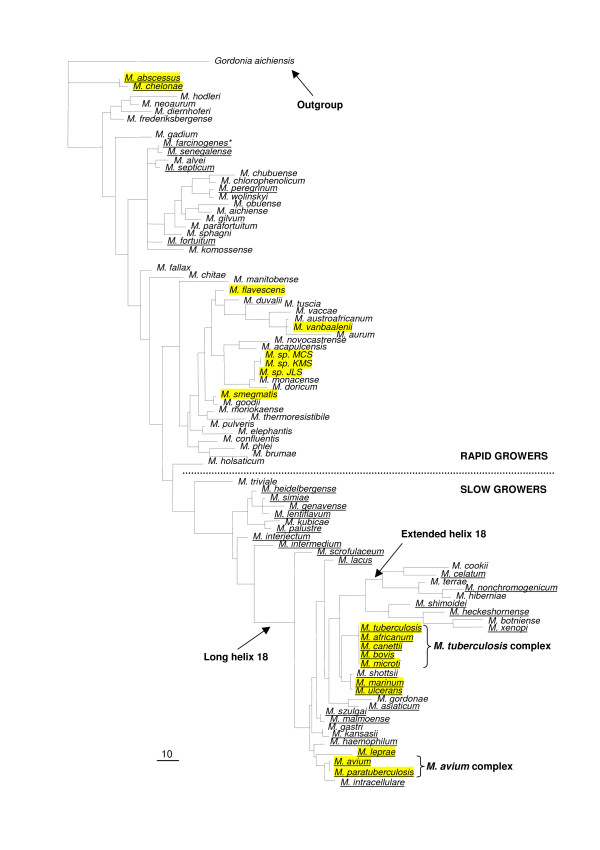
**Phylogenetic tree of all the members of the genus *Mycobacterium***. Strict consensus of the 230 most parsimonious trees using Paup 4.0b10 (heuristic search, gaps = fifth state) [89] from the 1286 aligned nucleotides of the 16S rRNA DNA sequence of 80 species of the genus *Mycobacterium *with the sequence of the species *Gordonia aichiensis *as the outgroup. Sequenced genomes are highlighted in yellow. The division between fast and slow-growing species is indicated by a dotted line. Underlined species are considered pathogens [62]. The members of the *M. tuberculosis *complex and the *M. avium *complex are indicated. The divisions between the normal helix 18, long helix 18 and extended helix 18 of the 16S rRNA gene sequence are indicated [94,95]. * = *M. farcinogenes *is a slow growing mycobacterium.

A phylogenetic tree was generated using the 16S rRNA gene sequence of 83 species of the genus *Mycobacterium*, with the sequence of the species *Gordonia aichiensis *as the outgroup (Figure 3). This was done in order to determine the evolutionary history of the genus *Mycobacterium *and to identify the sequenced species closest to the origin/last common ancestor of the genus. This species would provide the most valuable data with regards to the presence and origin of the ancestral PE and PPE genes. The taxonomical relationships between members of the genus *Mycobacterium *based on the 16S rRNA gene sequence information in this tree is comparable to data published previously by Pitulle *et al*. [61], Shinnick and Good [62] and Springer *et al*. [63]. The phylogenetic positions of all the sequenced mycobacterial species are indicated in yellow in Figure 3. From this analysis it is apparent that the non-pathogenic, fast-growing mycobacterium *M. smegmatis *is the sequenced species closest to the last common ancestor (the genome sequences of *M. abscessus *and *M. chelonae *have not been released publicly) and the genome sequence of this species thus represents the ancestral reference point for the investigation of the evolution of these gene families within the mycobacteria.

#### Comparative genomics analyses between *M. tuberculosis *H37Rv and *M. smegmatis*

Analysis of the genome sequence of *M. smegmatis *revealed only two pairs of the PE and PPE gene families. None of the other members of the PE or PPE gene families, including any of the PE_PGRS or PPE-MPTR genes, could be detected within the *M. smegmatis *genome. The first pair corresponds to the Rv3872/3 orthologues (MSMEG0062 and MSMEG0063) from ESAT-6 (*esx*) gene cluster region 1 (70% and 55% similarity to the *M. tuberculosis *H37Rv proteins, respectively), while the second pair corresponds to the Rv0285/6 orthologues (MSMEG0608 and MSMEG0609) from ESAT-6 (*esx*) gene cluster region 3 (87% and 64% similarity to the *M. tuberculosis *H37Rv proteins, respectively). These two gene pairs have been shown to be required for *in vivo*, and *in vitro *growth, respectively, in *M. tuberculosis *H37Rv [47,48]. Thus, the only PE and PPE genes present within the *M. smegmatis *genome are found within two ESAT-6 (*esx*) gene cluster regions.

#### The PE and PPE genes from ESAT-6 region 1 are the most ancestral genes of the two gene families

PE/PPE gene pairs are frequently associated with the ESAT-6 (*esx*) gene clusters in *M. tuberculosis *[1,64]. The duplication order of the ESAT-6 (*esx*) gene clusters within the genome of *M. tuberculosis *was previously predicted by systematic phylogenetic analyses of the constituent genes [1]. This duplication order was shown to extend from the ancestral region named region 4 (Rv3444c-Rv3450c) to region 1 (Rv3866-Rv3883c), 3 (Rv0282-Rv0292), 2 (Rv3884c-Rv3895c), and lastly to region 5 (Rv1782-Rv1798) (Figure 1A). The absence of a pair of PE and PPE genes within the most ancestral ESAT-6 region, region 4 (a region which is also present in species outside of the genus *Mycobacterium*)[1], indicates that these genes may have been integrated into the first duplicate of this region (region 1), and have subsequently been co-duplicated together with the rest of the genes within the subsequent four regions (Figure 1).

The genome of *M. smegmatis *only contains three of the five ESAT-6 (*esx*) gene cluster regions (regions 4, 1, and 3), with regions 2 and 5 being absent [1]. Although it is possible that regions 2 and 5 may have been deleted from the genome of this organism, it is more likely that they only evolved after the divergence of *M. smegmatis*, as these regions were determined to be the last two duplicates of the ESAT-6 (*esx*) gene cluster evolution [1]. This is supported by comparative genomics analyses of the genomes of closely-related fast-growing mycobacteria *M. flavenscens*, *M. vanbaalenii*, *M. sp *MCS and *M. sp *JLS in which ESAT-6 (*esx*) gene cluster regions 2 and 5 were also found to be absent, as well as *M. sp *KMS in which ESAT-6 (*esx*) gene cluster region 2 was present, but region 5 was absent (results not shown). This is further supported by the fact that the genome of *M. smegmatis *is approximately 1.7 times larger than that of *M. tuberculosis *[65], and thus does not display the same reductive properties to that observed in the genome of, for example, *M. leprae *(which was confirmed to have lost ESAT-6 (*esx*) gene cluster region 2 and 4 by deletion, [66]). As the only copies of the PE and PPE gene families found in the genome of *M. smegmatis *were present in ESAT-6 (*esx*) regions 1 and 3, and as the PE and PPE genes are not found outside of the genus *Mycobacterium*, it is clear that the members of the PE and PPE genes found within the ESAT-6 (*esx*) gene cluster regions 1 and 3 are the most ancestral representatives of these two gene families. Furthermore, as ESAT-6 (*esx*) gene cluster region 1 is the first duplicate of the ESAT-6 gene cluster regions, the PE and PPE gene copies from region 1 are probably the progenitors of all other PE and PPE genes. This is further supported by the observation that, although these two genes do contain the conserved N-terminal PE and PPE regions, respectively, they do not contain any long and complex C-termini as found in other representatives of the families, and thus represent a pre-C-terminal elongation and repeat-region formation stage.

### Phylogeny of the PE and PPE protein families in *M. tuberculosis *H37Rv

#### Phylogenetic analysis of the ancestral PE and PPE genes situated within the ESAT-6 (esx) gene clusters in *M. tuberculosis* H37Rv

**Figure 4:**
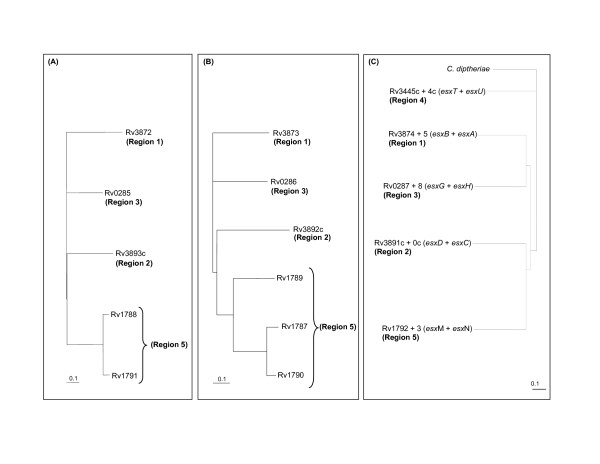
**Phylogeny of the PE and PPE protein families present within the ESAT-6 (*esx*) gene clusters in *M. tuberculosis *H37Rv**. Phylogenetic trees of the PE and PPE proteins, respectively, present within the ESAT-6 (esx) gene clusters in *M. tuberculosis *H37Rv, demonstrating a duplication order similar to that observed with other genes in the *M. tuberculosis *ESAT-6 (*esx*) gene cluster regions [1]. (A) PE proteins, (B) PPE proteins and (C) ESAT-6/CFP-10 proteins.

To confirm that the PE and PPE genes found within the ESAT-6 (*esx*) gene cluster regions in *M. tuberculosis *shared an evolutionary history with the other genes within the clusters (indicating co-duplication/evolution), we constructed separate phylogenetic trees based on the results of the independent analyses of the members of the PE and PPE families present in the 4 PE/PPE-containing ESAT-6 (*esx*) gene cluster regions (regions 1, 3, 2 and 5). The resulting phylogenetic trees (Figure 4) showed topologies congruent to those of phylogenetic trees obtained for all the other gene families situated in the ESAT-6 (*esx*) gene clusters [1]. From this we concluded that the PE and PPE genes were duplicated together with the ESAT-6 (*esx*) gene clusters after their initial insertion (into region 1), rather than being inserted during multiple separate subsequent events. These results also confirm the previously determined duplication order of the ESAT-6 (*esx*) gene clusters [1].

#### Phylogenetic analysis of all the PE and PPE genes present in *M. tuberculosis* H37Rv

**Figure 5:**
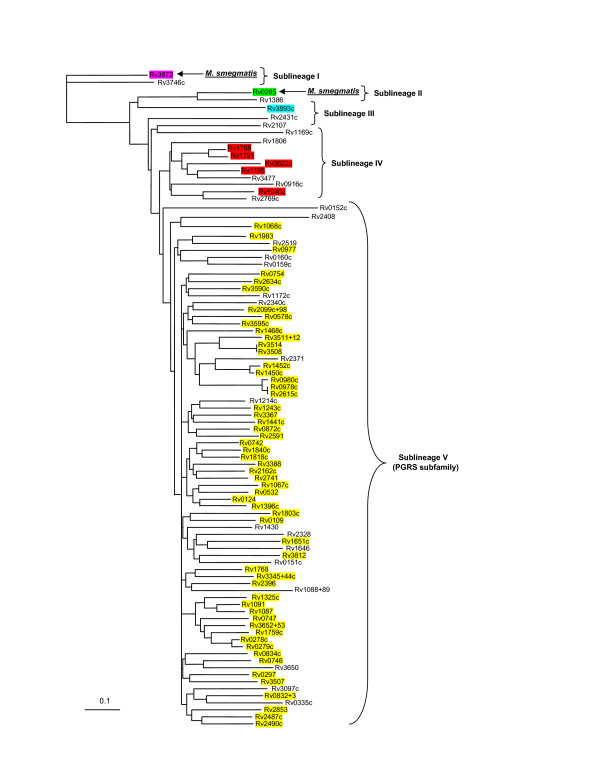
**Phylogenetic reconstruction of the evolutionary relationships between the members of the PE protein family**. The phylogenetic tree was constructed from the phylogenetic analyses done on the 110 aa N-terminal domains of the PE proteins. The tree was rooted to the outgroup, Rv3872 (PE35), shown to be the first PE insertion into the ESAT-6 (*esx*) gene clusters (region 1). The genes highlighted in purple, green and blue are present in ESAT-6 (*esx*) gene cluster region 1, 3 and 2, respectively. Genes highlighted in red are present in or have been previously shown to be duplicated from ESAT-6 (*esx*) gene cluster region 5 [1] and genes highlighted in yellow are members of the PGRS subfamily of the PE family. Arrows indicate orthologues of genes identified to be present within the *M. smegmatis *genome sequence. Five sublineages (including the PE_PGRS subfamily) are indicated by Roman numerals.

**Figure 6:**
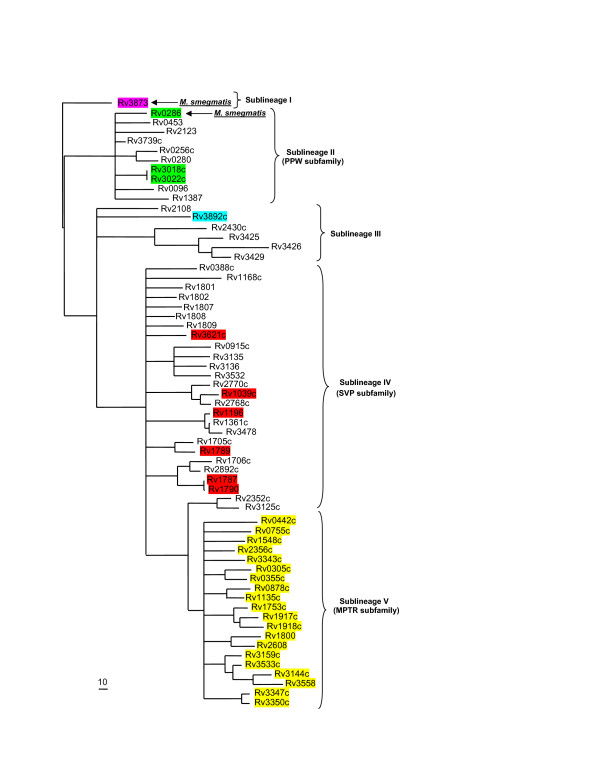
**Phylogenetic reconstruction of the evolutionary relationships between the members of the PPE protein family**. The phylogenetic tree was constructed from the phylogenetic analyses done on the 180 aa N-terminal domains of the PPE proteins. The tree was rooted to the outgroup, Rv3873 (PPE68), shown to be the first PPE insertion into the ESAT-6 (*esx*) gene clusters (region 1). The gene highlighted in purple is present in ESAT-6 (*esx*) gene cluster region 1, genes highlighted in green are present in or have been previously shown to be duplicated from ESAT-6 (*esx*) gene cluster region 3 [1], the gene highlighted in blue is present in ESAT-6 (*esx*) gene cluster region 2, genes highlighted in red are present in or have been previously shown to be duplicated from ESAT-6 (*esx*) gene cluster region 5 [1] and genes highlighted in yellow are members of the MPTR subfamily of the PPE family. Arrows indicate orthologues of genes present within the *M. smegmatis *genome sequence. Five sublineages (including the PPE-PPW, PPE-SVP and PPE-MPTR subfamilies) are indicated by Roman numerals.

To obtain a global picture of the evolutionary relationships of all PE and PPE genes within *M. tuberculosis *and not only those situated within the ESAT-6 (*esx*) gene clusters, we constructed independent phylogenetic trees based on the results of the multiple sequence alignments of all proteins encoded by members of the two gene families. The phylogenetic tree constructed from the ninety-six chosen PE protein family N-terminal sequences (see Methods) was rooted to the ancestral PE outgroup from ESAT-6 (*esx*) gene cluster region 1, namely Rv3872 (PE35, Figure 5). Similarly, the PPE protein from ESAT-6 (*esx*) gene cluster region 1, namely Rv3873 (PPE68), was chosen as the outgroup to root the phylogenetic tree constructed independently from the sixty-four PPE sequences (Figure 6). Both trees (from the PE and PPE families, respectively) showed a similar topology, which was conserved when the complete protein sequences were used for analysis instead of only the conserved N-termini (data not shown). Each tree was characterized by five distinct (but corresponding) sublineages (indicated by Roman numerals in Figure 5 and 6). Four of these sublineages match the PE_PGRS, PPE-PPW, PPE-SVP and PPE-MPTR subfamilies, respectively, and these results are thus in accordance with the subgroupings of the PE and PPE families proposed previously [12,14,16].

Since the tree topologies correspond to each other, it also suggests a co-evolutionary history for the two gene families. Interestingly, this evolutionary scenario is also congruent with the evolutionary history determined for the five ESAT-6 (*esx*) gene clusters, with duplication events of PE and PPE genes contained and associated with these regions expanding sequentially from region 1 to 3, 2 and lastly region 5. The topology of the phylogenetic trees suggests that the PE_PGRS and the PPE-MPTR subfamilies are the result of the most recent evolutionary events and have evolved from the sublineage that include the ESAT-6 (*esx*) gene cluster region 5 PE and PPE genes (Figure 5 and 6, sublineage IV). This is supported by the finding that some members (Rv1361c/PPE19, Rv3135/PPE50 and Rv3136/PPE51) of the PPE sublineage IV (PPE-SVP subfamily) contain isolated MPTR-like repeats, suggesting the existence of a common progenitor gene from which the PPE-MPTR subfamily expanded (data not shown). The proteins outside of the PE_PGRS and PPE-MPTR subfamilies, seem to be closer in homology to the ancestral genes, and are thus collectively called the "ancestral-type" PE and PPE genes for the purpose of discussion in this study.

The genes from ESAT-6 (*esx*) gene cluster region 5 seem to be highly prone to duplication, as region 5 is the only one of the five ESAT-6 (*esx*) gene clusters which contains multiple copies of the PE and PPE genes situated inside the cluster (Figure 1). Furthermore, ESAT-6 (*esx*) gene cluster region 5 is also the parent of a number of secondary duplications containing only the genes for PE, PPE, ESAT-6 (*esx*) and CFP-10 (a member of the *esx *family) (see Figure 1B and 1C) [1]. It appears that this region plays an important role in the propagation of both the ESAT-6/CFP-10 and the PE/PPE genes. It is thus tempting to speculate that the duplication propensity of the region 5 genes may have resulted in the initial subsequent expansion of the PGRS and MPTR subfamilies, although inherent properties of the PGRS and MPTR repeats themselves certainly also contributed to this phenomenon.

Closer inspection of the relative positions of the PE and PPE genes in the *M. tuberculosis *genome sequence revealed that in a number of cases a copy of each of these families was found situated adjacent to each other (Table 2, see also Tundup *et al*. [64] and Strong *et al*. [67]). By examining the relative positions of the PE and PPE genes from each pair on the separate PE and PPE phylogenetic trees, it was found that these pairs of genes are always situated in the same sublineage on the trees, indicating that they were likely to be co-duplicated. Furthermore, the order of their positions is always conserved, with the PE gene found situated upstream of the PPE gene. These paired genes are found in all the sublineages except in the highly polymorphic PGRS and MPTR subfamilies (sublineage V). In this sublineage, member genes were found situated on their own within a specific genomic location. Thus, it is clear that the expansion of the PGRS and MPTR subfamilies was associated with a change in their duplication characteristics, and although the cause and significance of this is unknown, it may point to a corresponding change in function. In support of this, in a computational identification of beta-barrel outer-membrane proteins of *M. tuberculosis*, Pajon *et al*. [26] identified 40 PE and PPE proteins from a total of 114 predicted beta-barrel structures. Closer inspection of the identified proteins indicate that they all form part of sublineage V, the PE_PGRS and PPE-MPTR subfamilies (23 and 17 members, respectively), indicating a shared function between the members of these two subfamilies.

**Table 2:** Paired genes present in both the PE and PPE multigene families.*

Sub-Lineage**	**Paired genes**		Associated ESAT-6 (*esx*) gene cluster region***
		
	**(PE)**	**(PPE)**	
I	Rv3872	Rv3873	Situated in ESAT-6 (*esx*) gene cluster region 1
II	Rv0285	Rv0286	Situated in ESAT-6 (*esx*) gene cluster region 3
II	Rv3018A	Rv3018c	Duplicated from ESAT-6 (*esx*) gene cluster region 3
II	Rv3022A	Rv3021/22c	Duplicated from ESAT-6 (*esx*) gene cluster region 3
II	Rv1386	Rv1387	Associated with ESAT-6 (*esx*) gene cluster region 3
III	Rv3893c	Rv3892c	Situated in ESAT-6 (*esx*) gene cluster region 2
III	Rv2107	Rv2108	Associated with ESAT-6 (*esx*) gene cluster region 2
III or IV	Rv2431c	Rv2430c	Associated with ESAT-6 (*esx*) gene cluster region 2 or 5
IV	Rv1788/91	Rv1787/89/90	Situated in ESAT-6 (*esx*) gene cluster region 5
IV	Rv3622c	Rv3621c	Duplicated from ESAT-6 (*esx*) gene cluster region 5
IV	Rv1195	Rv1196	Duplicated from ESAT-6 (*esx*) gene cluster region 5
IV	Rv1040c	Rv1039c	Duplicated from ESAT-6 (*esx*) gene cluster region 5
IV	Rv1169c	Rv1168c	Associated with ESAT-6 (*esx*) gene cluster region 5
IV	Rv1806	Rv1801/2/7/8/9	Associated with ESAT-6 (*esx*) gene cluster region 5
IV	Rv3477	Rv3478	Associated with ESAT-6 (*esx*) gene cluster region 5
IV	Rv2769c	Rv2768c/70c	Associated with ESAT-6 (*esx*) gene cluster region 5
IV	Rv0916c	Rv0915c	Associated with ESAT-6 (*esx*) gene cluster region 5

* Although they are physically-separated, Rv3746c and Rv3739c seems to have been a pair associated with either ESAT-6 gene cluster region 1 or 3, which was disrupted by the insertion of a number of genes.

** See Figure 5 and 6 for definition of sublineages.

*** "Situated in" denotes genes situated within ESAT-6 (*esx*) gene cluster regions, "Duplicated from" denotes genes confirmed to be duplicated from ESAT-6 (*esx*) gene clusters due to the presence of ESAT-6 and CFP-10 genes immediately adjacent to them, "Associated with" denotes genes which are hypothesized to have been duplicated from ESAT-6 (*esx*) gene cluster regions, as they are very homologous to their paralogues within the clusters (see Figure 1).

The reason for the maintenance of the gene pairing of the ancestral PE and PPE genes is still unclear, although these genes may be functionally related and co-transcribed. There is some early evidence for the latter from gene expression data obtained during adaptation to nutrient starvation (the gene pairs Rv0285/86 (PE5/PPE4), Rv1195/96 (PE13/PPE18), Rv1386/87 (PE15/PPE20) and Rv2431c/30c (PE25/PPE41) are downregulated and the pair Rv1169c/68c (PE11/PPE17) is upregulated [68]). Furthermore, it was recently demonstrated that the genes from at least one of these PE-PPE gene pairs, Rv2430c/31c, are co-transcribed and that the gene products interact with each other to form a hetero-tetramer [64]. This finding was expanded upon by Strong *et al*. [67], who determined the structure of the Rv2430c/31c protein interaction, and demonstrated that the PE/PPE protein pair forms a 1:1 complex. Intriguingly, this is similar to the situation observed for the proteins transcribed by the CFP-10 and ESAT-6 genes (adjacently situated to many of the PE-PPE gene pairs – see Figure 1A and 1B), which also forms a tight 1:1 complex [69-72] and is secreted by the ESAT-6 transport system [4-6,8]. There is evidence that the PPE protein encoded by Rv3873 (PPE68 from ESAT-6 (*esx*) gene cluster region 1) interacts with CFP-10, ESAT-6 and at least one other *esx *family member (Rv0288) [73]. It is thus tempting to speculate that the PE/PPE and *esx *genes are not only intricately linked phylogenetically, but also functionally, and that the PE/PPE complex may also be secreted by the ESAT-6 transport system. In support of this, Fortune *et al*. [10] have shown that the PE gene situated in ESAT-6 gene cluster region 1 (PE35 or Rv3872) are present (together with ESAT-6 and CFP-10 from ESAT-6 gene cluster region 1) in culture filtrates of *M. tuberculosis*.

Although a previous study by Espitia and colleagues aimed to address PE gene phylogeny, the authors had excluded 19 PE sequences from their phylogenetic calculations [44]. The absence of these sequences, which included the PE proteins belonging to the ESAT-6 (*esx*) gene cluster regions 1 (Rv3872/PE35), 2 (Rv3893c/PE36) and 3 (Rv0285/PE5), left a major gap in the study of the evolutionary expansion of this family. Our results differ from this study because we included these sequences, which have been shown in the current study to be the most ancestral representatives of the family, and thus form the roots from which the rest of the family expanded. We were thus able to root the tree and explain the evolutionary history of this gene family on the basis thereof.

### Comparative genomics analyses to verify the PE and PPE evolutionary history

In order to support the hypothesized evolutionary history deduced from the topologies of the PE and PPE phylogenetic trees generated in this study, we performed comparative genomics analyses of the sequenced genomes of *M. avium paratuberculosis*, *M. avium*, *M. leprae*, *M. ulcerans *and *M. marinum*, chosen as representative sequenced mycobacterial species phylogenetically situated between *M. smegmatis *and *M. tuberculosis *H37Rv (Figure 3).

#### *M. tuberculosis* H37Rv vs. *M. avium* and *M. avium paratuberculosis*

**Figure 7:**
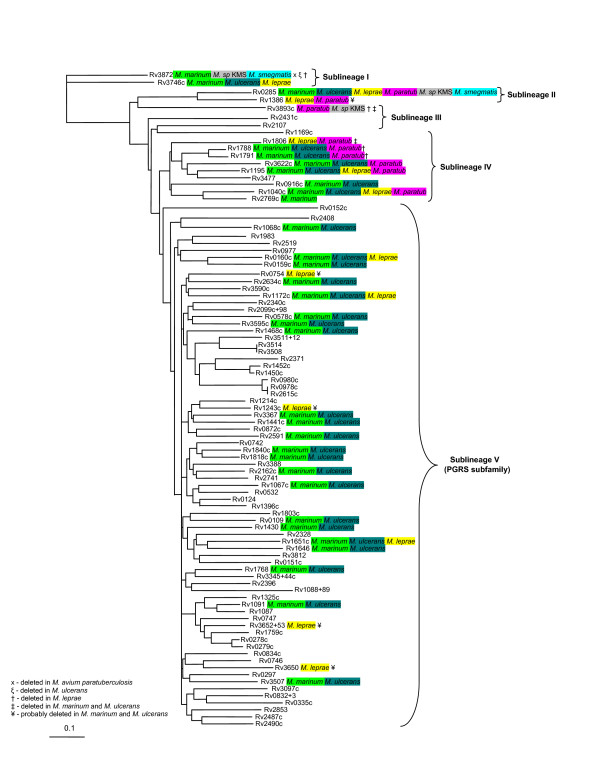
**Orthologues of *M. tuberculosis *PE genes present in the genomes of *M. smegmatis*, *M. sp*. KMS, *M. avium paratuberculosis, M. leprae, M. ulcerans *and *M. marinum***. PE genes identified in the genomes of *M. smegmatis *(highlighted in blue), *M. sp*. KMS (highlighted in grey), *M. avium paratuberculosis *(highlighted in purple), *M. leprae *(highlighted in yellow), *M. ulcerans *(highlighted in teal) and *M. marinum *(highlighted in green) are superimposed on the phylogenetic tree generated for the PE gene family in *M. tuberculosis *H37Rv (see Figure 5). *M. avium paratuberculosis*-, *M. leprae*-, *M. ulcerans*- and *M. marinum*-specific genes are omitted. *M. flavescens*, *M. vanbaalenii*, *M. sp*. MCS and *M. sp*. JLS PE genes show a similar distribution to *M. smegmatis *and are thus not indicated on the figure.

**Figure 8:**
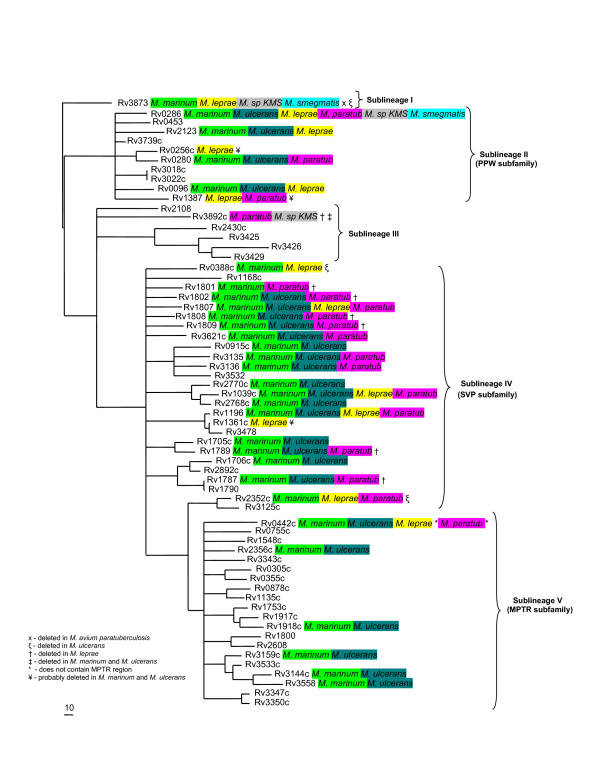
**Orthologues of *M. tuberculosis *PPE genes present in the genomes of *M. smegmatis, M. sp*. KMS, *M. avium paratuberculosis, M. leprae, M. ulcerans *and *M. marinum***. PPE genes identified in the genomes of *M. smegmatis *(highlighted in blue), *M. sp*. KMS (highlighted in grey), *M. avium paratuberculosis *(highlighted in purple), *M. leprae *(highlighted in yellow), *M. ulcerans *(highlighted in teal), and *M. marinum *(highlighted in green) are superimposed on the phylogenetic tree generated for the PPE gene family in *M. tuberculosis *H37Rv (see Figure 6). *M. avium paratuberculosis*-, *M. leprae*-, *M. ulcerans*- and *M. marinum*-specific genes are omitted. *M. flavescens*, *M. vanbaalenii*, *M. sp*. MCS and *M. sp*. JLS PPE genes show a similar distribution to *M. smegmatis *and are thus not indicated on the figure.

The results from the analysis between the genomes of *M. tuberculosis *H37Rv and *M. avium paratuberculosis *is summarized in Table 3. We found a total of 10 "ancestral-type" PE genes in the genome of *M. avium paratuberculosis *(compared to the 34 "ancestral-type" PE's in *M. tuberculosis*), of which one is *M. avium paratuberculosis*-specific. We could not find any genes belonging to the PE_PGRS subfamily, consistent with the observation by Li *et al*. [74]. We also identified 37 PPE genes in the genome of *M. avium paratuberculosis *(compared to the 69 in *M. tuberculosis*), of which only one (NT03MA4150, an orthologue of Rv0442c/PPE10) belongs to the PPE-MPTR subfamily, and 18 are *M. avium paratuberculosis*-specific. When these results were superimposed on the phylogenetic trees generated for the PE and PPE gene families in *M. tuberculosis *H37Rv (Figures 7 and 8, respectively, *M. avium paratuberculosis*-specific genes were omitted), they showed clearly that all the members of the PE and PPE gene families that are present in the genome of *M. avium paratuberculosis *form part of the "ancestral-type" genes, except for the orthologue of Rv0442c. This supports the notion that these "ancestral-type" genes represent the earliest members of the PE and PPE gene families, and shows that the PE_PGRS and PPE-MPTR subfamilies have evolved only after the divergence of *M. avium paratuberculosis*. These results were compared with that obtained with the unfinished genome sequence database of *M. avium *104, which were found to correspond to what is observed in the *M. paratuberculosis *subspecies (data not shown). This also confirmed previously published hybridization analyses which showed the absence of PGRS sequences in the genome of *M. avium *[15,75].

**Table 3:** *M. avium paratuberculosis *PE and PPE genes*

**PE genes**		
**TIGR gene number**	**Primary gene number**	***M. tuberculosis *orthologue gene number**

NT03MA0124	MAP0122	Rv1386
NT03MA1039	MAP1003c	Rv1040c (included in the same sequence as Rv1039c – see below)
NT03MA0159	MAP0157	Rv3893c
NT03MA0465	MAP0441	Rv3622c
Not annotated (NT03MA1570.1)	-	Rv1788
NT03MA1572	MAP1507	Rv1791
NT03MA1580	MAP1514	Region 5
NT03MA2703	MAP2576c	Probably Rv1195, but have been rearranged
NT03MA3983	MAP3781	Rv0285
NT03MA4378	MAP4144	Absent in *M. tuberculosis *– situated between Rv0685 and Rv0686 – most homologous to PE Rv3595c

**PPE genes**		

**TIGR gene number**	**Primary gene number**	***M. tuberculosis *orthologue gene number**

NT03MA0125	MAP0123	Rv1387
NT03MA0160	MAP0158	Rv3892c
NT03MA0467	MAP0442	Rv3621c
NT03MA0998	MAP0966c	Absent in *M. tuberculosis *– Situated between Rv1006 and Rv1007 next to a transposase – most homologous to the PPE Rv1789
NT03MA1039	MAP1003c	Rv1039c (also includes Rv1040c in the same sequence)
NT03MA1194	MAP1144c	Absent in *M. tuberculosis *– Situated between Rv1417 and Rv1420-most homologous to the PPE Rv0280
NT03MA1201	MAP1152	Absent in *M. tuberculosis *– Situated between Rv1423 and Rv1425 next to a transposase – most homologous to the PPE Rv1808 and Rv1801
NT03MA1202	MAP1153	Absent in *M. tuberculosis *– Situated between Rv1423 and Rv1425-most homologous to the PPE Rv1809
NT03MA1204	MAP1155	Absent in *M. tuberculosis *– Situated between Rv1423 and Rv1425-most homologous to the PPE Rv1807
NT03MA1570	MAP1505	Rv1787
NT03MA1571	MAP1506	Rv1789
NT03MA1581	MAP1515	Region 5 – most homologous to Rv1807
NT03MA1582	MAP1516	Region 5 – most homologous to Rv1807
NT03MA1585	MAP1518	Region 5 – most homologous to Rv1808
NT03MA1586	MAP1519	Region 5 – most homologous to Rv1809 and Rv1802
NT03MA1589	MAP1521	Region 5 – most homologous to Rv1808 and Rv1801
NT03MA1590	MAP1522	Region 5 – most homologous to Rv1809
NT03MA1746	MAP1675	Absent in *M. tuberculosis *– Situated between large number of *M. avium *genes absent from *M. tuberculosis *– most homologous to PPE Rv3621c
NT03MA1809	MAP1734	Absent in *M. tuberculosis *– Situated between large number of *M. avium *genes absent from *M. tuberculosis *– most homologous to PPE Rv2123
NT03MA1810	-	Absent in *M. tuberculosis *– Situated between large number of *M. avium *genes absent from *M. tuberculosis *– most homologous to PPE Rv0280
NT03MA1895	MAP1813c	Absent in *M. tuberculosis *– Situated between Rv2066 and Rv2069 – most homologous to PPE Rv0256c
NT03MA2236	MAP2136c	Situated between Rv2348 and Rv2357c, in other words in the position of PPE Rv2352, Rv2353 or Rv2356, but does not contain MPTR tail and shows most homology to PPE Rv1789
NT03MA2702	MAP2575c	Probably Rv1196, but have been rearranged
NT03MA2725	MAP2595	Absent in *M. tuberculosis *– Situated between Rv1186c and Rv1185c – most homologous to PPE Rv0256c
NT03MA2730	MAP2600	Absent in *M. tuberculosis *– Situated between Rv1185c and Rv1181 – most homologous to PPE Rv1807
NT03MA2731	MAP2601	Absent in *M. tuberculosis *– Situated between Rv1185c and Rv1181 – most homologous to PPE Rv1808
NT03MA3070	MAP2927	Absent in *M. tuberculosis *– Situated between Rv2856 and Rv2857c – most homologous to PPE Rv2892c
NT03MA3359	MAP3184	Rv3135
NT03MA3360	MAP3185	Rv3136
NT03MA3611	MAP3419c	Absent in *M. tuberculosis *– Situated between Rv3298c and Rv3300c – most homologous to PPE Rv1789
NT03MA3612	MAP3420c	Absent in *M. tuberculosis *– Situated between Rv3298c and Rv3300c – most homologous to PPE Rv1809
NT03MA3690	MAP3490	Absent in *M. tuberculosis *– Situated between Rv3396c and Rv3400 – most homologous to PPE Rv0280
NT03MA3934	MAP3725	Rv0280
NT03MA3944	MAP3737	Absent in *M. tuberculosis *– Situated between large number of *M. avium *genes absent from *M. tuberculosis *– most homologous to PPE Rv0256c
NT03MA3967	MAP3765	Absent in *M. tuberculosis *– Situated between large number of *M. avium *genes absent from *M. tuberculosis *– most homologous to PPE Rv0280
NT03MA3984	MAP3782	Rv0286
NT03MA4150	MAP3939c	Rv0442c – Situated between Rv0441c and Rv0443, in other words in the position of PPE Rv0442c, but does not contain MPTR tail although it shows most homology to PPE Rv0442c

*The genes NT03MA3679 and NT03MA4076 was annotated as PE_PGRS family proteins in the TIGR annotation, but they are in fact not. NT03MA3679 is an orthologue of Rv3390 (lpqD), while NT03MA4076 is a gene which is absent in *M. tuberculosis *(situated between Rv0358 and Rv0357c). Although it is slightly homologous to the PE_PGRS Rv0754, this homology is only to bp 280 – 520 of the 584 bp PE_PGRS sequence (this region of homology is also highly homologous to lpqD and consists of a biphosphatase and phosphoglycerate mutase signature). It also does not contain the conserved PE N-terminus and is thus highly unlikely to be a PE_PGRS member.

**Figure 9:**
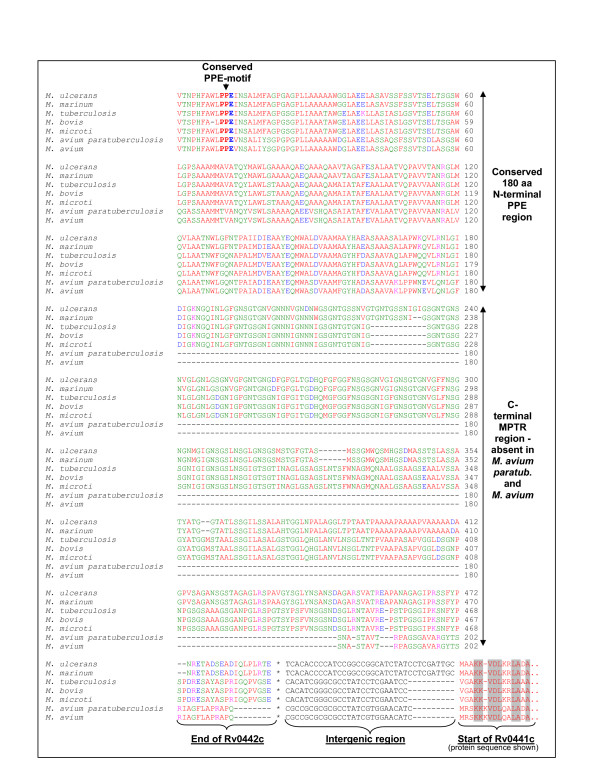
**Sequence alignments of orthologues of Rv0442c (PPE10) present in *M. ulcerans, M. marinum, M. tuberculosis, M. bovis, M. microti, M. avium paratuberculosis *and *M. avium***. Complete sequence alignment with orthologues from the genomes of all available species, showing conserved N-terminal regions and absence of MPTR region after base pair 180 in the *M. avium *complex. C-terminal region of Rv0442c protein is indicated, showing intergenic DNA region and start of the 3' flanking protein Rv0441c, proving that absence of the MPTR region is not due to wrong annotation of sequenced genomes. Homologous regions of the N-terminal part of Rv0441c are shaded in grey.

One of the most interesting results from the *M. avium paratuberculosis *analysis was the identification of NT03MA4150, an orthologue of the MPTR subfamily gene Rv0442c, the only MPTR orthologue identified in the genome of *M. avium paratuberculosis*. Closer inspection of the sequence of this and surrounding genes showed that this gene is a true orthologue of the *M. tuberculosis *MPTR gene Rv0442c (i.e. situated between the orthologues of Rv0441c and Rv0443, with the highest level of homology to Rv0442c). However, this gene in *M. avium paratuberculosis *does not contain the polymorphic MPTR C-terminal region characteristic of the MPTR subfamily and found in Rv0442c in *M. tuberculosis*. To confirm the result, a complete sequence alignment was done with the protein sequences of the orthologues of this gene from the genomes of all available mycobacterial species (Figure 9). From this analysis it was clear that members of the *M. avium *complex (*M. avium paratuberculosis *and *M. avium *104) do not contain the MPTR region in this gene, while members of species closer to *M. tuberculosis *(*M. marinum, M. ulcerans, M. bovis and M. microti*) do contain the repeat region. The homology between the orthologues of the *M. avium *complex and that of the other species end at exactly amino acid 180 (the consensus end for the conserved N-terminal region of the members of the PPE family). Furthermore, the tail region could not have been omitted from the annotation of the genome of *M. avium paratuberculosis*, as the 3' flanking gene (orthologue of Rv0441c) follows 27 bp after the stopcodon of NT03MA4150 (the intergenic region is 26 bp in *M. tuberculosis*, see Figure 9). This suggests that Rv0442c represents the first member of the MPTR subfamily to have been duplicated, before the acquisition of the MPTR repeat region. It is perhaps possible that *M. avium *and *M. avium paratuberculosis *could have lost all the genes belonging to the PE_PGRS and PPE-MPTR subfamilies, however, this is highly unlikely, as we could find no evidence of residues of genes or the presence of pseudogenes which could indicate a loss of function and degeneration.

#### *M. tuberculosis* H37Rv vs. *M. leprae*

To gain insight into the events taking place in the phylogenetic gap between the *M. tuberculosis *complex and the *M. avium *complex, we performed a comparative genomics analysis between the completed genome sequences of *M. tuberculosis *H37Rv and *M. leprae*. The genome sequence of *M. leprae *is known to have undergone extensive loss of synteny, inversion and genome downsizing [66], which may have resulted from recombination between dispersed copies of repetitive elements [76]. This has caused the loss of hundreds of genes, resulting in a genome littered with pseudogenes in various stages of decay and elimination. Our primary aim was thus not to identify the reason for the absence of members of the PE and PPE gene families (which could either be due to the fact that they were never present/duplicated, or that they were deleted), but rather to identify whether members were present (in an intact form), and if not, whether there were any residues left of members (pseudogenes) which may have been lost in the process of genome downsizing. Table 4 provides a summary of the members of the PE and PPE gene families present in the genome of *M. leprae*. We identified 14 genes from the "ancestral-type" PE family, of which 9 were pseudogenes and 5 were *M. leprae*-specific. In addition, 8 members of the PGRS subfamily could be identified in *M. leprae *(of which 7 were pseudogenes and 4 were *M. leprae*-specific), indicating that the expansion of the PGRS subfamily must have started before the divergence of this organism (Figure 7 – *M. leprae*-specific genes were omitted). It is interesting to note that, although there were 8 detectable PGRS members, 7 of them were pseudogenes and only one intact PGRS gene could be identified in this species, consistent with previously published hybridization studies which showed a general absence of PGRS sequences in the genome of *M. leprae *[15]. Analysis of the PPE subfamily led to the identification of 26 members of the "ancestral-type" (of which 19 were pseudogenes and 13 were *M. leprae*-specific), with no MPTR subfamily members present, except for ML2369c, the orthologue of Rv0442c/PPE10 (which is also the only representative present in the genomes of *M. avium *and *M. avium paratuberculosis*). In Figure 8, members of the PPE family identified in this study were superimposed on the phylogenetic tree generated for the PPE gene family in *M. tuberculosis *H37Rv (*M. leprae*-specific genes were omitted). With the exception of the orthologue of Rv0442c (ML2369c), no residues or pseudogenes of any of the other MPTR subfamily genes present in *M. tuberculosis *H37Rv could be identified in the genome of *M. leprae *(including the *M. leprae*-specific genes). This suggests that the MPTR subfamily was not duplicated in the genome of this organism, and that the expansion of the MPTR subfamily thus occurred after the divergence of *M. leprae*. Although it is possible that the extensive genome downsizing in *M. leprae *could have caused the loss of all the members of this gene subfamily, it is highly unlikely, and no evidence for this was observed (no pseudogenes or residues of genes were found as in the case of the PGRS subfamily).

**Table 4:** *M. leprae *PE, PGRS and PPE genes

**PE genes**			
***M. leprae *gene number**	*M. leprae *gene name*	***M. tuberculosis *orthologue gene number**	***M. tuberculosis *orthologue gene name**

ML0196c (pseudogene)	PE 1	Rv3650	PE33
ML0263c (pseudogene)	PE2	Rv1040c	PE8
ML0410	PE3	*M. leprae*-specific	-
ML0538	PE4	Rv1386	PE15
ML1053	PE5	Rv1195	PE13
ML1183c	PE6	*M. leprae*-specific paralogue of Rv1195	-
ML1493 (pseudogene)	PE7	Rv1172c	PE12
ML1534c (pseudogene)	PE8	Rv1806	PE20
ML1743c (pseudogene)	PE9	*M. leprae*-specific	-
ML2534c	PE10	Rv0285	PE5
ML2632 (pseudogene)	PE11	Rv0160c	PE4
ML1865c (pseudogene)	-	*M. leprae*-specific	-
ML2129c (pseudogene)	-	*M. leprae*-specific	-
ML 2477 (pseudogene)	-	Rv3746c	PE34

**PE_PGRS genes**			

***M. leprae *gene number**	*M. leprae *gene name*	***M. tuberculosis *orthologue gene number**	***M. tuberculosis *orthologue gene name**

ML0194c (pseudogene)	PE_PGRS1	Rv3653	PE_PGRS61
ML0495 (pseudogene)	PE_PGRS2	*M. leprae*-specific	-
ML1092c (pseudogene)	PE_PGRS3	Rv1243c	PE_PGRS23
ML1403 (pseudogene)	PE_PGRS4	Rv1651c	PE_PGRS30
ML1414 (pseudogene)	PE_PGRS5	*M. leprae*-specific	-
ML2241c (pseudogene)	PE_PGRS6	Rv0754	PE_PGRS11
ML0147 (pseudogene)	-	*M. leprae*-specific	-
ML0946	-	*M. leprae*-specific	-

**PPE genes**			

***M. leprae *gene number**	*M. leprae *gene name*	***M. tuberculosis *orthologue gene number**	***M. tuberculosis *orthologue gene name**

ML0026 (pseudogene)	PPE1	*M. leprae*-specific	-
ML0277 (pseudogene)	PPE2	Rv0388c	PPE9
ML0328c (pseudogene)	PPE3	*M. leprae*-specific paralogue of Rv3021c	paralogue of PPE47
ML0503 (pseudogene)	PPE4	*M. leprae*-specific	-
ML0539	PPE5	Rv1387	PPE20
ML0797c (pseudogene)	PPE6	*M. leprae*-specific	-
ML0828 (pseudogene)	PPE7	Rv2352c	PPE38
ML1054 (pseudogene)	PPE8	Rv1196	PPE18
ML1182c	PPE9	*M. leprae*-specific paralogue of Rv1196	-
ML1308c (pseudogene)	PPE10	Rv2123	PPE37
ML1533c (pseudogene)	PPE11	Rv1807	PPE31
ML1828c	PPE12	Rv0256c	PPE2
ML1991	PPE13	Rv0096	PPE1
ML2369c (pseudogene)	PPE14	Rv0442c	PPE10
ML2533c (pseudogene)	PPE15	Rv0286	PPE4
ML2538c (pseudogene)	PPE16	*M. leprae*-specific, situated between Rv0281 and 82	-
ML0051c	PPE68	Rv3873	PPE68
ML0411	-	*M. leprae*-specific	-
not annotated, situated between ML0262c and 63c (pseudogene)	-	Rv1039c	PPE15
ML1754 (pseudogene)	-	Rv1361c	PPE19
ML0588	-	*M. leprae*-specific	-
ML1935c (pseudogene)	-	*M. leprae*-specific	-
ML1967c (pseudogene)	-	*M. leprae*-specific	-
ML1968c (pseudogene)	-	*M. leprae*-specific	-
ML2128c (pseudogene)	-	*M. leprae*-specific	-
ML2243c (pseudogene)	-	*M. leprae*-specific	-

* Regrettably, and in contrast to the annotation of the PE and PPE genes in *M. bovis*, the genes in *M. leprae *are annotated according to their own PE and PPE numbers and not according to the same numbers as its corresponding orthologue in *M. tuberculosis*, with the only exception being PPE68 which have the same name in both species.

To confirm the absence of MPTR genes in this species, we analyzed the sequence of ML2369c (the Rv0442c orthologue) to determine whether it contains the C-terminal MPTR region which is present in Rv0442c in *M. tuberculosis*, but absent in the Rv0442c orthologues of *M. avium *and *M. avium paratuberculosis*. Although the gene is a pseudogene and has undergone extensive degradation at the C-terminus, complicating the sequence alignment, it is clear that there are no MPTR repeats present in this region, even when the C-terminal region is translated into any of the three potential open reading frames (data not shown). This suggests that *M. leprae *diverged after the start of the expansion of the PGRS subfamily, but before that of the MPTR's.

#### *M. tuberculosis* H37Rv vs. *M. ulcerans* and *M. marinum*

*M. ulcerans *and *M. marinum *are phylogenetically closely-related and are also phylogenetically close relatives of the members of the *M. tuberculosis *complex (see Figure 3). The genomes of both of these organisms have been sequenced, with the *M. ulcerans *Agy99 genome annotation completed and the *M. marinum *M genome sequence in the process of being annotated (Table 1). These genome sequences thus provide an excellent resource to determine the status of the expansion of the MPTR subfamily of the PPE gene family in two species situated immediately outside of the *M. tuberculosis *complex.

An analysis of the genome of *M. ulcerans *was carried out to determine the presence and absence of orthologues of the members of the PE and PPE gene families of *M. tuberculosis *H37Rv in this organism. The results from the analysis between *M. tuberculosis *H37Rv and *M. ulcerans *are summarized in Additional file 1. We identified 21 genes from the "ancestral-type" PE family in the genome of *M. ulcerans *(compared to the 34 in *M. tuberculosis*), of which 6 were pseudogenes and 8 were *M. ulcerans*-specific. Of the 6 pseudogenes, 4 were *M. ulcerans*-specific. In addition, 121 members of the PE_PGRS subfamily could be identified in *M. ulcerans *(compared to the 65 in *M. tuberculosis*), of which 66 were pseudogenes and 104 were *M. ulcerans*-specific. Of the 66 pseudogenes, 59 were *M. ulcerans*-specific. Analysis of the PPE subfamily led to the identification of 81 members (compared to the 69 in *M. tuberculosis*) of which 34 were pseudogenes and 55 were *M. ulcerans*-specific. Of the 34 pseudogenes, 25 were *M. ulcerans*-specific. Six orthologues of members of the *M. tuberculosis *PPE-MPTR subfamily were present in the genome of *M. ulcerans*, including the orthologue of Rv0442c/PPE10 (MUL_1395), in this case containing an MPTR repeat region (see also Figure 9). Interestingly, 5 of these 6 PPE-MPTR orthologues were pseudogenes, with the only intact subfamily member being the orthologue of Rv0442c, although 9 intact *M. ulcerans*-specific PPE-MPTR subfamily members were also detected (MUL_0782, MUL_0890, MUL_0893, MUL_0902, MUL_0964, MUL_0965, MUL_2586, MUL_0098 and MUL_3169). These results are superimposed on the phylogenetic trees generated for the PE and PPE gene families in *M. tuberculosis *H37Rv in Figures 7 and 8 (*M. ulcerans*-specific genes are omitted). This suggests that the acquisition of the MPTR repeat region in the C-terminus of Rv0442c and the expansion of the MPTR subfamily took place before the divergence of *M. ulcerans*. *M. ulcerans *also had a vast specific expansion of the PE and PPE families, resulting in 55 more genes belonging to these two gene families than in *M. tuberculosis *H37Rv, although a large number of them have become pseudogenes, resulting in a lesser number of functional genes in *M. ulcerans *(117 genes) compared to *M. tuberculosis *H37Rv (168 genes). It is interesting to note that the majority of the pseudogenes from these two gene families in the genome of *M. ulcerans *are *M. ulcerans*-specific copies (88 out of 106 pseudogenes), and may thus represent "unsuccessful evolutionary experiments".

An analysis of the genome of *M. marinum *was carried out to determine the presence and absence of orthologues of the members of the PE and PPE gene families of *M. tuberculosis *H37Rv in this organism, in order to confirm the observations of the *M. ulcerans *genome. As the genome sequence of *M. marinum *is still in the annotation phase, no gene names or numbers are available, but the results of the analyses are superimposed on the phylogenetic trees generated for the PE and PPE gene families in *M. tuberculosis *H37Rv in Figure 7 and 8 (*M. marinum*-specific genes are omitted). The results are analogous to what was observed in *M. ulcerans *(confirming their relatedness), and shows the presence of multiple copies of both the PGRS and MPTR subfamilies. This confirms the previously published hybridization data which indicated the presence of multiple copies of the PGRS sequence in the genome of *M. marinum *[15]. There are, analogous to *M. ulcerans*, also 6 orthologues of members of the *M. tuberculosis *PPE-MPTR subfamily present in the genome of *M. marinum*, one of which is the orthologue of Rv0442c, in this case also containing an MPTR repeat region (see Figure 9). This supports the observation of the *M. ulcerans *genome sequence and confirms that the acquisition of the MPTR repeat region in the C-terminus of Rv0442c and the expansion of the MPTR subfamily took place before the divergence of *M. marinum *and *M. ulcerans*.

### Comparative genomics for extent of sequence variation

To further examine the relationships between, and evolutionary history of, the members of the subfamilies of the PE and PPE protein families, to identify subfamily-specific characteristics, and to determine the extent of PE and PPE sequence similarity and variation, orthologues in the fully sequenced and annotated genomes of *M. tuberculosis *H37Rv and CDC1551 were analyzed by comparative genomics. During this analysis, a complete investigation of the presence and absence of genes, gene sizes, frameshifts, insertions and deletions (indels), alternative start sites, protein mismatches and conservative substitutions was performed. Although other strains of *M. tuberculosis *are also being sequenced (including strains 210, A1, Ekat-4, K, F11, C, Haarlem, Peruvian1, Peruvian2 and W-148 – see Table 1), these sequences are not completed and verified and thus not useful for an analysis where, for example, single nucleotide polymorphisms are investigated. Additional file 2 provides an overview of the reasons for size differences between annotated genes from the two genome databases. This analysis shows that the "ancestral-type" members of both the PE and PPE families, and specifically the members present within the ESAT-6 (*esx*) gene cluster regions, have remained conserved between the two different strains (with the only reason for a difference in size being artificial, due to the use of an alternative start site during genome annotation). This is in contrast to the members of the PGRS and MPTR subfamilies, which show considerable variation in size due to frameshifts, insertions and deletions. Additional file 3 shows a summary of the extent of sequence variation on a protein level between the orthologues of these gene families in the two *M. tuberculosis *strains and from this it is clear that the "ancestral-type" PE and PPE genes are highly conserved between strains, while the MPTR and especially the PGRS subfamilies are more prone to sequence variation (the only exception to this is PPE60 which is not an MPTR but shows a high level of variation between the strains). These variations mostly occur in the C-terminal polymorphic domain (after the conserved N-terminal domain of approximately 110 amino acids for the PE members, and 180 amino acids for the PPE members), clearly demonstrating the importance of the conservation of the N-terminal domain. The results from this study are in agreement with previously-published results by Garnier and coworkers [30], who found blocks of sequence variation in genes encoding 29 different PE_PGRS and 28 PPE proteins (most of which belong to the PPE_MPTR subfamily) resulting from frameshifts, insertions and deletions in a comparison between the annotated genes from the completed genomes of *M. bovis *AF2122/97 and *M. tuberculosis *H37Rv. The authors speculate that this indicates that these families can support extensive sequence polymorphism and could thus provide a potential source of antigenic variation. It is thus possible that the members of the PGRS and MPTR subfamilies have evolved to function as a source of antigenic variation; a function which probably differs from the original function still performed by the members of the "ancestral-type" subgroup (including the members present within and associated with the ESAT-6 (*esx*) gene cluster regions). The genome sequencing of other members of the *M. tuberculosis *complex which are currently being performed (*M. microti*, *M. africanum*, and *M. canettii*) will undoubtedly shed more light on the variation observed between the orthologues of these two large polymorphic subfamilies.

### Presence of the PPE-MPTR's in members of the genus *Mycobacterium*

**Figure 10:**
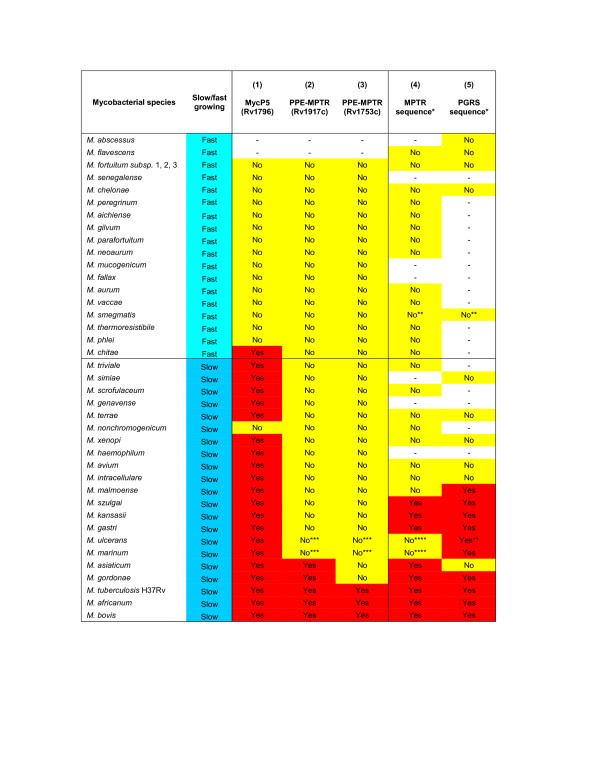
**Southern hybridization analyses of the genomic DNA of 37 different species of the genus *Mycobacterium***. Summary of Southern blot results obtained with mycosin 5 (column 1) and PPE-MPTR probes (column 2 and 3) in comparison to previously-published results using MPTR and PGRS sequences, respectively (column 4 and 5), as indicated. Presence of hybridization signal is indicated by the word "Yes", while absence of signal is indicated by "No". The sign "-" indicates that hybridization was not performed in this species. Mycobacterial species are separated into fast- and slow-growing species (see Figure 3). * MPTR and PGRS hybridization results were obtained from previously-published studies by Hermans *et al*. [17], Ross *et al*. [75] and Poulet *et al*. [15]. ** data obtained from whole genome sequence information – see Table 1. *** negative results for Rv1917c and Rv1753c in *M. marinum *and *M. ulcerans *is in agreement with the genome sequencing data which indicated the absence of both of these genes within the genomes of this species. **** although previously published data indicated a failure of the MPTR repeat sequence to hybridize to the genomic DNA of these species, *M. marinum*- and *M. ulcerans*-specific PPE-MPTR genes have been identified in the current study through genome sequencing data.

In order to confirm the exclusive expansion of the PPE-MPTR subfamily in the genomes of members of the *M. tuberculosis *complex and species closely-related to it, we performed Southern blot analyses of different mycobacterial species using two selected PPE-MPTR gene probes (Table 5), and compared this to previously published data on the distribution of the MPTR repeat sequence. A probe for the mycosin gene *mycP5 *(Rv1796), was also selected to be used as a marker for the presence or absence of ESAT-6 (*esx*) gene cluster region 5 within the genomes of these different species. The mycosins are a family of subtilisin-like serine proteases found within the ESAT-6 (*esx*) gene cluster regions (Figure 1) [1,77,78] and represent the most conserved genes within the ESAT-6 (*esx*) cluster regions when orthologues of different species are compared (data not shown). The Southern blot analysis was done with genomic DNA of species of both the fast- and slow-growing mycobacterial groups (see Figure 3 and Table 6) and the results are summarized in Figure 10.

**Table 5:** Primer sequences used to generate probes

**Probe name**	**Primer name**	**Primer sequence (5' to 3')**	**Application**
Rv1917c (and Rv1918c)	ppe-17	ttc aac tcc gtg acg tcg	*Amplification of 471 bp 5' terminal region from Rv1917c and Rv1918c*
	ppe-18	cag cac acc ctt gga act g	
Rv1753c	1753ISH_F	cgg tgg ctt tag tct acc tgc	*Amplification of 279 bp 5' terminal region from Rv1753c*
	1753ISH_R	ccg gtc aat gtg tat ggg tg	
*mycP5 *(Rv1796)	prot 5 f	gtg ctc gta atg tca tcg	*Amplification of 658 bp of Rv1796*
	prot 5 r	cat atc ggc acc ata tcg	

**Table 6:** Mycobacterial species used to obtain genomic DNA

**Mycobacterial species**	**Slow/fast growing**	**ATCC number**
*M. africanum*	Slow	ATCC 25420
*M. aichiense*	Fast	ATCC 27280
*M. asiaticum*	Slow	ATCC 25276
*M. aurum*	Fast	ATCC 23366
*M. avium*	Slow	ATCC 25291
*M. bovis*	Slow	ATCC 19210
*M. chelonae*	Fast	ATCC 35749
*M. chitae*	Fast	ATCC 19627
*M. fallax*	Fast	ATCC 35219
*M. fortuitum*	Fast	ATCC 6841
*M. fortuitum*	Fast	ATCC 49403
*M. fortuitum*	Fast	ATCC 49404
*M. gastri*	Slow	ATCC15754
*M. genavense*	Slow	ATCC 51233
*M. gilvum*	Fast	ATCC 43909
*M. gordonae*	Slow	ATCC 14470
*M. haemophilum*	Slow	ATCC 29548
*M. intracellulare*	Slow	ATCC 13950
*M. kansasii*	Slow	ATCC 12478
*M. malmoense*	Slow	ATCC 29571
*M. marinum*	Slow	ATCC 927
*M. mucogenicum*	Fast	ATCC 49650
*M. neoaurum*	Fast	ATCC 25795
*M. nonchromogenicum*	Slow	ATCC 19530
*M. parafortuitum*	Fast	ATCC 19686
*M. peregrinum*	Fast	ATCC 14467
*M. phlei*	Fast	ATCC 11758
*M. scrofulaceum*	Slow	ATCC 19981
*M. senegalense*	Fast	ATCC 35796
*M. simiae*	Slow	ATCC 25275
*M. smegmatis*	Fast	ATCC 19420
*M. szulgai*	Slow	ATCC 35799
*M. terrae*	Slow	ATCC 15755
*M. thermoresistibile*	Fast	ATCC 19527
*M. triviale*	Slow	ATCC 23292
*M. tuberculosis *H37Rv	Slow	ATCC 25618
*M. ulcerans*	Slow	ATCC 19423
*M. vaccae*	Fast	ATCC 15483
*M. xenopi*	Slow	ATCC 19250

The first analysis was done using the probe for *mycP5*, the mycosin present in ESAT-6 (*esx*) gene cluster region 5. This probe gave an indication of the distribution of the ESAT-6 (*esx*) gene cluster region 5 within the genomes of other mycobacterial species, as region 5 was hypothesized in this study to be the origin of both the SVP and MPTR subfamilies of the PPE gene family. The results showed that the ESAT-6 (*esx*) gene cluster region 5 was only present within the genomes of the slow-growing mycobacterial species tested. The only exception for this is the slow-growing species *M. nonchromogenicum*, which might have undergone a deletion of this region. No hybridization was found with any members of the fast-growing group except for *M. chitae*, indicating either that the ESAT-6 (*esx*) gene cluster region 5 is absent from the genomes of these species, or that the species are evolutionarily so far removed from the slow-growers that the gene homology was insufficient to allow hybridization under the stringent conditions used in the analysis. Given the absence of region 5 in the genomes of *M. smegmatis*, *M. flavescens*, *M. vanbaalenii*, *M. sp*. KMS, *M. sp*. MCS and *M. sp*. JLS, it is highly likely that this region is absent from all fast-growing species and that these species have diverged before the duplication of region 5.

In order to obtain insight into the expansion and distribution of the PPE-MPTR subfamily within the slow-growing mycobacterial species, we used the two genes Rv1917c (PPE34) and Rv1753c (PPE24) as representatives of the PPE-MPTR sublineage (V) for Southern hybridization analysis. The hybridization signals were specific and appeared to be restricted to specific members of the slow growing mycobacterial group within and surrounding the *M. tuberculosis *complex, namely *M. gordonae, M. asiaticum, M. tuberculosis*, *M. bovis *and *M. africanum *(in the case of Rv1917c) and *M. tuberculosis*, *M. bovis *and *M. africanum *in the case of Rv1753c (Figure 10). The fact that both Rv1917c and Rv1753c did not hybridize to *M. marinum *and *M. ulcerans *is in agreement with the genome sequencing data which indicated the absence of both of these genes within the genomes of these species. The results also confirms the absence of these genes in the genomes of the members of the *M. avium *complex. Furthermore, the results compared favorably to previously published data (see Column 4, Figure 10) in which the MPTR repeat region probe was used for hybridization, and in which only species situated in the *M. tuberculosis *complex, or closely-related to the complex, were identified [17].

Previously published hybridization data on the PGRS repeat sequence [15,75] also confirms the broader distribution and earlier expansion of this subfamily in comparison to the PPE-MPTR subfamily within the slow-growing members of the genus *Mycobacterium *(see Column 5, Figure 10). This data supports the evolutionary history proposed in this study with the expansion of the PGRS subfamily (after the divergence of the *M. avium *complex) preceding that of the MPTR subfamily.

In summary, the hybridization results support the proposed phylogenetic relationships of the gene families, and are likely to reflect evolutionary divergence/branch points of different mycobacterial species, interspersed by periods of PE/PPE/ESAT-6 duplication and expansion.

## Conclusion

**Figure 11:**
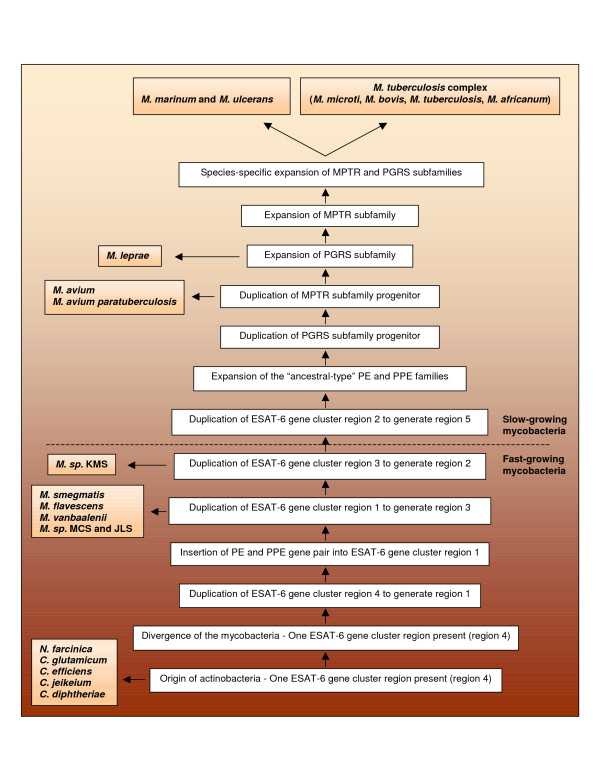
**Reconstruction of the evolutionary history of the PE and PPE gene families of the genus *Mycobacterium***. Schematic representation of the suggested evolutionary history of the PE and PPE gene families. The results of this study indicated that these genes were initially inserted into the ESAT-6 (*esx*) gene cluster region 1 after the duplication of the cluster, and have subsequently been duplicated along with the ESAT-6 regions. The expansion of the PE and PPE gene families have occurred in unison with the expansion of the ESAT-6 (*esx*) gene family, throughout the evolution of the genus. Members of the genus *Mycobacterium *investigated in this study, have diverged at the positions indicated. After each main duplication event involving a complete ESAT-6 (*esx*) gene cluster region, a number of secondary subduplications of the PE and PPE genes (in some cases associated with a copy of the ESAT-6 and CFP-10 genes, occurred from the newly duplicated ESAT-6 (*esx*) gene cluster region. The highly duplicated PE_PGRS and PPE-MPTR subfamilies originated after the divergence of the *M. avium *complex and *M. leprae*, respectively. Both families were present before the divergence of *M. marinum*/*M. ulcerans *and the *M. tuberculosis *complex.

Phylogenetic reconstruction of the evolutionary history of the PE and PPE gene families suggests that the first pair of these genes were initially inserted into the ESAT-6 (*esx*) gene cluster region 1, and have subsequently been duplicated along with the regions (Figure 11). After each main duplication event involving a complete ESAT-6 (*esx*) gene cluster region, a number of secondary subduplications of the PE and PPE genes (in some cases associated with a copy of the ESAT-6 and CFP-10 genes, [1]) occurred from the newly duplicated ESAT-6 (*esx*) gene cluster region. This phenomenon is predicted to have culminated in the duplication of the ESAT-6 (*esx*) gene cluster region 5, from which a large number of PE and PPE genes (the so-called SVP subfamily of the PPE gene family) were duplicated separately to the rest of the genome. Furthermore, the evolutionary history predicted by the phylogenetic trees suggests that the highly duplicated PE_PGRS subfamily and subsequently the PPE-MPTR subfamily have originated from a duplication from ESAT-6 (*esx*) gene cluster region 5. It thus seems as if the PE and PPE genes present within region 5 have an enhanced propensity for duplication, their mobility driving the expansion of these genes into the highly polymorphic PGRS and MPTR subfamilies, respectively.

The data presented in the study suggests that the PE_PGRS subfamily expansion preceded the emergence of the PPE-MPTR subfamily. A possible explanation for this observation comes from the fact that there are some resemblance between the MPTR repeat sequence (GCCGGTGTTG) and the complementary sequence of the core region of two PGRS repeat elements arranged in tandem (TT**GCCGCCGTTG**CCGCCG) [15,17]. This may indicate a potential role for the C-terminal PGRS repeat of the PE gene family in the emergence of the C-terminal MPTR element of the PPE gene family, and may point to an evolutionary event through insertion/recombination between the two gene families and subsequent expansion in the MPTR subfamily. In support of this, Adindla and Guruprasad [16] have identified three PPE-MPTR proteins (Rv1800/PPE28, Rv3539/PPE63 and Rv2608/PPE42) which showed sequence similarity to five PE proteins (Rv1430/PE16, Rv0151/PE1, Rv0152/PE2, Rv0159/PE3 and Rv0160/PE4) corresponding to a 225 amino acid C-terminal region, which they named the "PE-PPE domain". Although not identified as true PGRS-containing PE genes, all five these genes form part of sublineage V (the PGRS-containing sublineage) and may therefore represent precursors to the PE_PGRS sequences. There are thus some genes from the PE and MPTR subfamilies which share levels of homology in their C-termini. This is further supported by the data from Pajon *et al*. [26] which showed that a large proportion of the members from the PE_PGRS and PPE-MPTR subfamilies share beta-barrel outer-membrane protein structures, and that one of these outer-membrane anchoring domains consists of the proposed conserved "PE-PPE domain" identified by Adindla and Guruprasad [16].

A number of recent studies using diverse approaches have shown that the ESAT-6 (*esx*) gene clusters encode a novel secretory apparatus [1-5,50] Most recently, the demonstration by Okkels *et al*. [24] that Rv3873 (PPE68), the PPE gene present in the RD1 region, is a potent T-cell antigen, lead these authors to speculate that the ESAT-6 (*esx*) gene cluster promotes the presentation of key antigens, including members of the PE and PPE protein families, to the host immune system. It is tempting to speculate that the ESAT-6/CFP-10 loci together with their associated PE/PPE genes represent what might be thought of as an "immunogenicity island". Further studies are under way to determine whether the ESAT-6 (*esx*) gene cluster regions are able to secrete members of the PE and PPE protein families, whether this secretion is specific for members of the "ancestral-type" group found in the cluster regions, and whether the recently-evolved PGRS/MPTR types can also use this secretion system.

The large number of genes within the PE and PPE gene families has confounded past attempts to choose representative members of the families for further analysis. This study provides a logical starting point by defining the evolutionary history of the gene families, and elucidating the relationships and specific features of the different subgroups. An informed choice concerning candidate genes for further study can now be made, based on position of the member on the evolutionary tree, association or not with the ESAT-6 gene clusters, and subgroup-specific features. In this way, studies based upon a random choice of members, which may be biased in not being representative of the whole spectrum of different members within these families, could be avoided. It also provides the opportunity to study subgroups instead of individual members, to determine what functional differences, if any, exists between these different subgroups.

In conclusion, we aimed to investigate the evolutionary history of the PE and PPE gene families in relation to their observed association with four of the five ESAT-6 (*esx*) gene cluster regions. We have demonstrated that the expansion of the PE and PPE families is linked to the duplications of the ESAT-6 (*esx*) gene clusters. We have also shown that this association has led to the absence of multiple duplications of the PE and PPE families, including the total absence of the multigene PE_PGRS and PPE-MPTR subfamilies, in the fast-growing mycobacteria, including *M. smegmatis*. We have shown that the expansion of the PE_PGRS and PPE-MPTR subfamilies took place after the divergence of the *M. avium *complex, and that the PGRS and the MPTR expansions started before the divergence of *M. leprae *and *M. marinum*, respectively. This study contributes to the understanding of the PE and PPE gene families, in terms of stability, absence/presence of the PE and PPE genes within the genomes of various mycobacteria, and their association with the ESAT-6 (*esx*) gene clusters. The results of this study also provides for a logical starting point for the selection of candidates for further study of these large multigene families.

## Methods

### Genome sequence data and comparative genomics analyses

Annotations, descriptions, gene and protein sequences of individual genes belonging to the PE and PPE families were obtained from the publicly available finished and unfinished genome sequence databases of the organisms listed in Table 1. For comparative genomics, the genome sequence databases were compared to that of *M. tuberculosis *H37Rv, in order to identify orthologous genes. BLAST similarity searches [79] using the respective *M. tuberculosis *H37Rv protein sequences and the tblastn algorithm were performed using the WU-BLAST version 2.0 (Gish, W. 1996–2005 – [80]) server in the database search services of the TIGR [81], Sanger Centre [82] and Genolist (Pasteur Institute) [83] websites. To confirm the identity of the resulting sequences, open reading frames adjacent to the identified genes were examined to determine if they matched the genes surrounding the corresponding *M. tuberculosis *PE and PPE genes, thereby confirming the identity of the orthologue. The unfinished genome sequences were examined in a similar manner, but were not analyzed in detail as sequencing is still incomplete.

### Phylogenetic tree of all the members of the genus *Mycobacterium*

The 16S rRNA gene sequences of 83 species of the genus *Mycobacterium*, as well as the species *Gordonia aichiensis*, was used to generate a phylogenetic tree of the genus *Mycobacterium*. All species were selected from the Ribosomal Database Project-II Release 9 [84] to be type strains containing only near-full-length 16S rRNA sequences (>1200 bases, no short partials), except for the species *M. chelonae*, *M. spagni*, *M. abscessus*, *M. confluentis*, *M. genavense*, *M. interjectum*, *M. intermedium*, *M. marinum*, *M. ulcerans*, *M. haemophilum*, *M. acapulcensis*, *M. lentiflavum*, *M. pulveris*, *M. manitobense*, *M. monacense*, *M. brumae*, and *M. moriokaense*, which did not have any type strains with a near-full-length sequence of longer than 1200 bases available in the database. For some of these species (*M. abscessus*, *M. confluentis*, *M. marinum*), sequences from type strains from the German Collection of Microorganisms and Cell Cultures (DSM) [85] were available and could thus be used. For the rest, representatives with sequences of longer than 1200 bases were chosen according to correct alignment with type strains. The following strains were chosen for all species (type strain indicated by the letter T in brackets after the name):*M. abscessus *(T); DSM 44196, *M. acapulcensis*; ATCC 14473, *M. aichiense *(T); ATCC 27280, *M. alvei *(T); CIP 103464, *M. asiaticum *(T); ATCC 25276, *M. aurum *(T); ATCC 23366, *M. austroafricanum *(T); ATCC 33464, *M. avium *subsp. *paratuberculosis *(T); ATCC 19698, *M. botniense *(T); E347, *M. brumae*; ATCC 51384, *M. celatum *(T); L08169, *M. chelonae*; ATCC 35752, *M. chitae *(T); ATCC 19627, *M. chlorophenolicum *(T); PCP-I, *M. chubuense *(T); ATCC 27278, *M. confluentis *(T); DSM 44017, *M. cookii *(T); ATCC 49103 (T) = NZ2., *M. diernhoferi *(T); ATCC 19340, *M. doricum *(T); FI-13295, *M. duvalii *(T); ATCC 43910, *M. elephantis *(T); AJ010747, *M. fallax *(T); M29562, *M. farcinogenes *(T); ATCC35753, *M. flavescens *(T); ATCC 14474, *M. fortuitum *(T); ATCC 6841, *M. frederiksbergense *(T); DSM 44346, *M. gadium *(T); ATCC 27726, *M. gastri *(T); ATCC 15754, *M. genavense *X60070, *M. gilvum *(T); ATCC 43909, *M. goodii *(T); M069, *M. gordonae *(T); ATCC 14470, *M. haemophilum *X88923, *M. heckeshornense *(T); S369, *M. heidelbergense *(T); 2554/91, *M. hiberniae *(T); ATCC 9874, *M. hodleri *(T); DSM 44183, *M. holsaticum *(T); 1406, *M. interjectum *X70961, *M. intermedium *X67847, *M. intracellulare *(T); ATCC 15985, *M. kansasii *(T); M29575, *M. komossense *(T); ATCC 33013, *M. kubicae *(T); CDC 941078, *M. lacus *(T); NRCM 00-255, *M. lentiflavum*; ATCC 51985, *M. leprae *(T); X53999, *M. malmoense *(T); ATCC 29571, *M. manitobense*; NRCM 01-154, *M. marinum *(T); DSM 44344, *M. monacense*; B9-21-178, *M. moriokaense*; DSM 44221T, *M. neoaurum *(T); M29564, *M. nonchromogenicu*m (T); ATCC 19530, *M. novocastrense *(T); 73, *M. obuense *(T); ATCC 27023, *M. palustre *(T); E846, *M. parafortuitum *(T); DSM 43528, *M. peregrinum *(T); ATCC14467, *M. phlei *(T); M29566, *M. pulveris*; DSM 44222T, *M. scrofulaceum *(T); ATCC 19981, *M. senegalense *(T); M29567, *M. septicum *(T); W4964, *M. shimoidei *(T); ATCC 27962, *M. shottsii *(T); M175, *M. simiae *(T); ATCC 25275, *M. smegmatis *(T); ATCC 19420, *M. sp. KMS*; AY083217, *M. sp. MCS*; CP000384, *M. sp. JLS*; AF387804, *M. sphagni*; ATCC 33026, *M. szulgai *(T); ATCC 25799, *M. terrae *(T); ATCC 15755, *M. thermoresistibile *(T); M29570, *M. triviale *(T); ATCC 23292, *M. tuberculosis *(T); H37/Rv, *M. tusciae *(T); FI-25796, *M. ulcerans *X58954, *M. vaccae *(T); ATCC 15483, *M. vanbaalenii *(T); DSM 7251 = PYR-1, *M. wolinskyi *(T); 700010, *M. xenopi *(T); M61664, *G._aichiensis *(T); ATCC 33611T. Multiple sequence alignments of these gene sequences were done using ClustalW 1.8 on the WWW server at the European Bioinformatics Institute website [86,87]. The alignments were manually checked for errors and refined where appropriate using BioEdit version 5.0.9. [88]. The final tree was taken as the strict consensus of the 230 most parsimonious trees generated using Paup 4.0b10 (heuristic search, gaps = fifth state) [89] from the 1286 aligned nucleotides of the 16S rRNA DNA sequence of the 83 species of the genus *Mycobacterium*, with the sequence of the species *Gordonia aichiensis *as the outgroup.

### Clean-up and generation of PE and PPE datasets

The phylogenetic reconstruction of the evolutionary relationships of the members of the PE and PPE protein families of *M. tuberculosis *H37Rv was done by analyses of four separate datasets. Clean-up of sample sets involved preliminary alignment to check for sequence instability or misalignments, as well as confirmation of gene annotation by comparative analyses. The first two datasets included the protein sequences of all the members of the PE and PPE protein families, respectively, that are present within the four ESAT-6 (*esx*) gene clusters in the genome of *M. tuberculosis *H37Rv.

The third dataset comprised the protein sequences of the sixty-nine members of the PPE family in the *M. tuberculosis *H37Rv database. Eleven of the predicted PPE proteins did not contain the characteristic N-terminal PPE motif. However, in six of these (Rv0305c/PPE6, Rv3425/PPE57, Rv3426/PPE58, Rv3429/PPE59, Rv3539/PPE63 and Rv3892c/PPE69) this was only due to a substitution in one of the two proline residues in the conserved motif. These six protein sequences could thus be reliably aligned to the rest of the family members due to a high percentage of sequence homology and were included in the dataset. The other five proteins (Rv0304c/PPE5, Rv0354c/PPE7, Rv2353c/PPE39, Rv3021c/PPE47 and Rv3738c/PPE66) were excluded from the analysis as it was found that their upstream regions were disrupted by either IS*6110 *insertion or apparent frameshift mutations, and they could thus not be aligned for phylogenetic analyses.

The fourth dataset contained the protein sequences of the ninety-nine members of the PE family in the *M. tuberculosis *H37Rv database. One of the members of the predicted PE family (Rv3020c) was found [1] to have been annotated incorrectly as a PE by Cole *et al*. [12], while two other members (Rv3018A/PE27A and Rv2126c/PE_PGRS37) could not be reliably aligned due to a loss of the N-terminal conserved region, and all three were thus excluded from further analyses. Six members (Rv0833/PE_PGRS13, Rv1089/PE10, Rv2098c/PE_PGRS36, Rv3344c/PE_PGRS49, Rv3512/PE_PGRS56, and Rv3653PE_PGRS61), which also did not have conserved N-termini, were shown to be situated adjacent to a gene encoding for the N-terminus (Rv0832/PE_PGRS12, Rv1088/PE9, Rv2099c/PE21, Rv3345c/PE_PGRS50, Rv3511/PE_PGRS55, and Rv3652/PE_PGRS60, respectively). Closer inspection of this organization suggested that each of these gene pairs in fact represented one gene that was split by stopcodon formation during frameshifting. Thus, each pair of proteins from this group were combined and included as one protein sequence in the analyses. Stopcodons were left out of these combined sequences.

### Multiple sequence alignments

Due to the highly polymorphic nature of the C-terminal region of the PE and PPE proteins, the conserved N-terminal domains of 100 aa and 180 aa for the PE and PPE proteins, respectively, were initially used to construct the multiple sequence alignments. Multiple sequence alignments of the protein sequences of the ninety-six PE and sixty-four PPE proteins were done using ClustalW 1.8 on the WWW server at the European Bioinformatics Institute website [86,87]. The alignments were manually checked for errors and refined where appropriate. Subsequent alignments using the complete sequences (containing both conserved N- and polymorphic C-terminal regions) were done to confirm results obtained with only conserved N-termini.

### Phylogenetic trees

Phylogenetic analyses were done using the neighbor-joining algorithm in the program PAUP 4.0b10 [89], and 1000 subsets were generated for Bootstrapping resampling of the data. Confidence intervals for the internal topology of the trees were obtained from the resampling analyses and only nodes occurring in over 50% of the trees were assumed to be significant [90]. All branches with a zero branch length were collapsed. Based on the evolutionary order defined for the ESAT-6 (*esx*) gene clusters [1] and the results from the analysis of the genome sequence of *M. smegmatis*, we have used the ancestral PE and PPE genes present within ESAT-6 (*esx*) gene cluster region 1 (Rv3872/PE35 and Rv3873/PPE68, respectively) as the outgroups to assign as roots. The consensus trees of the above were calculated using the majority rule formula and were drawn using the program Treeview 1.5 [91].

### Comparative genomics for extent of sequence variation

To determine the extent of PE and PPE sequence variation and elucidate the differences between orthologues of subfamilies of these gene families in the genomes of *M. tuberculosis *H37Rv and CDC1551, a complete comparative analysis of the presence and absence of genes, gene sizes, frameshifts, insertions and deletions (indels), alternative start sites, protein mismatches and conservative substitutions was done.

### Primers and probes

The primers used to generate probes for Southern hybridization to genomic DNA are listed in Table 5. PPE-MPTR and *mycP *probes were generated using the selected primers to individually PCR amplify regions from the PPE-MPTR genes Rv1917c (PPE34) and Rv1753c (PPE24), as well as from the mycosin gene *mycP5 *(Rv1796).

### Southern hybridization

Genomic DNA was isolated from different mycobacterial species (obtained from the American Type Culture Collection (ATCC), see Table 6) as previously described [92]. Genomic DNA was digested with *Alu*I or *BstE*II, electrophoretically fractionated, Southern transferred and hybridized as previously described [93]. Probing of Southern blots was done using selected ECL-labeled probes as listed in Table 5.

## List of Abbreviations

PE - protein family characterized by Proline-Glutamic Acid motif

PPE - protein family characterized by Proline-Proline-Glutamic Acid motif

PGRS - "polymorphic GC-rich-repetitive sequence" subfamily of the PE family

MPTR - "major polymorphic tandem repeat" subfamily of the PPE family

SVP - subfamily of the PPE family characterized by the motif Gly-X-X-Ser-Val-Pro-X-X-Trp

PPW - subfamily of the PPE family characterized by the motifs Gly-Phe-X-Gly-Thr and Pro-X-X-Pro-X-X-Trp

indels - insertions or deletions

ESAT-6 - 6 kDa Early Secreted Antigenic Target (*esx*)

CFP-10 - 10 kDa Culture Filtrate Protein

## Authors' contributions

NCGvP conceived of and designed the study, carried out the sequence alignments, comparative genomics and phylogenetics, interpreted the results and drafted the manuscript. SLS helped conceive of the study, participated in its design, carried out sequence alignments and was involved in interpretation of the results and drafting of the manuscript. HL and YK carried out the DNA extractions and Southern hybridizations. PDvH and RMW participated in the design and coordination of the study, were involved in interpreting the results and helped to draft the manuscript. All authors read and approved the final manuscript.

## Supplementary Material

Additional file 1 *M. ulcerans *PE, PGRS and PPE genes. The data provided represent presence and absence of all orthologues of the members of the PE and PPE gene families of *M. tuberculosis *H37Rv in *M. ulcerans *(this file is the *M. ulcerans *equivalent to the data that is presented for *M. avium paratuberculosis *and *M. leprae *in Tables 3 and 4). Click here for file

Additional file 2 Comparative genomics for gene size differences between *M. tuberculosis *H37Rv and CDC1551. The data in this table provide an overview of the reasons for size differences observed between annotated PE and PPE genes from the two *M. tuberculosis *genome databases, indicating that variation in size due to frameshifts, insertions and deletions is largely associated with the PE_PGRS and PPE-MPTR subfamilies. Click here for file

Additional file 3 Comparative genomics for extent of sequence variation between *M. tuberculosis *H37Rv and CDC1551. The data in this table provide an overview of the extent of sequence variation on a protein level between the orthologues of the PE and PPE families in the two *M. tuberculosis *strains, indicating that the "ancestral-type" PE and PPE genes are highly conserved between strains, while the PPE-MPTR and PE_PGRS subfamilies are prone to sequence variation. Click here for file
