# Supplementary material for: Evolution and expansion of the Mycobacterium tuberculosis PE and PPE multigene families and their association with the duplication of the ESAT-6 (esx) gene cluster regions
Source: BMC Evol Biol. 2006 Nov 15;6:95. doi: 10.1186/1471-2148-6-95 (PMC1660551; doi:10.1186/1471-2148-6-95)
Supplement: Additional file 1 — M. ulcerans PE, PGRS and PPE genes. The data provided represent presence and absence of all orthologues of the members of the PE and PPE gene families of M. tuberculosis H37Rv in M. ulcerans (this file is the M. ulcerans equivalent to the data that is presented for M. avium paratuberculosis and M. leprae in Tables 3 and 4). [file 1471-2148-6-95-S1.doc]

| **Additional file 1** | | | |
| --- | --- | --- | --- |
| ***M. ulcerans* PE, PGRS and PPE genes** | | | |
| **PE genes** | | | |
| ***M. ulcerans* gene number** | ***M. ulcerans* gene name** | ***M. tuberculosis* orthologue gene number** | ***M. tuberculosis* orthologue gene name** |
| MUL_0590 (pseudogene) | - | *M. ulcerans*-specific | - |
| MUL_0946* | PE31 | Rv1195 | PE13 |
| MUL_0967 | - | *M. ulcerans*-specific | - |
| MUL_1019 | - | Rv1172c | PE12 |
| MUL_1033 (pseudogene) | - | Rv0159c | PE3 |
| MUL_1034 | PE4 | Rv0160c | PE4 |
| MUL_1207 | PE5 | Rv0285 | PE5 |
| MUL_1632 |  | Rv1646 | PE17 |
| MUL_1822 (pseudogene) | PE16 | Rv1430 | PE16 |
| MUL_2246 | - | *M. ulcerans*-specific | - |
| MUL_2395 | PE26 | *M. ulcerans*-specific | - |
| MUL_2589 (pseudogene) |  | *M. ulcerans*-specific | - |
| MUL_3084 | PE19 | Rv1791 | PE19 |
| MUL_3088 | PE19_1 | Rv1788 | PE18 |
| MUL_3845 (pseudogene) | - | *M. ulcerans*-specific | - |
| MUL_4196 | PE32 | Rv3622c | PE32 |
| MUL_4336 | PE5_1 | *M. ulcerans*-specific | - |
| MUL_4359 | PE34 | Rv3746c | PE34 |
| MUL_4462 | - | Rv0916c | PE7 |
| MUL_4620 | PE8 | Rv1040c | PE8 |
| MUL_4726 (pseudogene) | - | *M. ulcerans*-specific | - |
| **PE_PGRS genes** | | | |
| ***M. ulcerans* gene number** | ***M. ulcerans* gene name** | ***M. tuberculosis* orthologue gene number** | ***M. tuberculosis* orthologue gene name** |
| MUL_0085 (pseudogene) | - | *M. ulcerans*-specific | - |
| MUL_0088 (pseudogene) | PE_PGRS50 | *M. ulcerans*-specific | - |
| MUL_0190 | PE_PGRS20 | Rv1091 | PE_PGRS22 |
| MUL_0212 | PE_PGRS9_3 | Rv1068c | PE_PGRS20 |
| MUL_0213 (pseudogene) | - | Rv1067c | PE_PGRS19 |
| MUL_0222 (pseudogene) | - | *M. ulcerans*-specific | - |
| MUL_0355 | - | *M. ulcerans*-specific | - |
| MUL_0391 | - | *M. ulcerans*-specific | - |
| MUL_0466 (pseudogene) | - | *M. ulcerans*-specific | - |
| MUL_0537 (pseudogene) | - | *M. ulcerans*-specific | - |
| MUL_0569 | - | *M. ulcerans*-specific | - |
| MUL_0570 | - | *M. ulcerans*-specific | - |
| MUL_0572 | - | *M. ulcerans*-specific | - |
| MUL_0694 (pseudogene) | - | Rv0578c | PE_PGRS7 |
| MUL_0740 | - | *M. ulcerans*-specific | - |
| MUL_0748 | - | *M. ulcerans*-specific | - |
| MUL_0973 (pseudogene) | - | *M. ulcerans*-specific | - |
| MUL_0978 (pseudogene) | - | *M. ulcerans*-specific | - |
| MUL_1013 (pseudogene) | - | *M. ulcerans*-specific | - |
| MUL_1014 (pseudogene) | - | *M. ulcerans*-specific | - |
| MUL_1015 (pseudogene) | - | *M. ulcerans*-specific | - |
| MUL_1021 | - | *M. ulcerans*-specific | - |
| MUL_1081 (pseudogene) | - | *M. ulcerans*-specific | - |
| MUL_1128 (pseudogene) | - | *M. ulcerans*-specific | - |
| MUL_1145 | PE_PGRS30 | *M. ulcerans*-specific | - |
| MUL_1149 | - | *M. ulcerans*-specific | - |
| MUL_1155 | - | *M. ulcerans*-specific | - |
| MUL_1326 (pseudogene) | - | *M. ulcerans*-specific | - |
| MUL_1339 | - | *M. ulcerans*-specific | - |
| MUL_1363 | - | *M. ulcerans*-specific | - |
| MUL_1387 (pseudogene) | - | *M. ulcerans*-specific | - |
| MUL_1448 (pseudogene) | - | *M. ulcerans*-specific | - |
| MUL_1450 | - | *M. ulcerans*-specific | - |
| MUL_1453 (pseudogene) | - | *M. ulcerans*-specific | - |
| MUL_1637 (pseudogene) | - | *M. ulcerans*-specific | - |
| MUL_1639 | - | Rv1651c | PE_PGRS30 |
| MUL_1640 | - | *M. ulcerans*-specific | - |
| MUL_1641 (pseudogene) | - | *M. ulcerans*-specific | - |
| MUL_1663 (pseudogene) | - | *M. ulcerans*-specific | - |
| MUL_1672 (pseudogene) | - | *M. ulcerans*-specific | - |
| MUL_1675 (pseudogene) | - | *M. ulcerans*-specific | - |
| MUL_1717 (pseudogene) | PE_PGRS44 | Rv2591 | PE_PGRS44 |
| MUL_1718 | - | *M. ulcerans*-specific | - |
| MUL_1721 | - | *M. ulcerans*-specific | - |
| MUL_1837 | - | *M. ulcerans*-specific | - |
| MUL_1838 | - | Rv1441c | PE_PGRS26 |
| MUL_1868 (pseudogene) | - | *M. ulcerans*-specific | - |
| MUL_1870 | - | Rv1468c | PE_PGRS29 |
| MUL_2099 (pseudogene) | - | *M. ulcerans*-specific | - |
| MUL_2172 (pseudogene) | - | *M. ulcerans*-specific | - |
| MUL_2207 (pseudogene) | - | *M. ulcerans*-specific | - |
| MUL_2250 | - | *M. ulcerans*-specific | - |
| MUL_2354 | - | *M. ulcerans*-specific | - |
| MUL_2485 | PE_PGRS9 | *M. ulcerans*-specific | - |
| MUL_2590 (pseudogene) | - | *M. ulcerans*-specific | - |
| MUL_2684 (pseudogene) | - | *M. ulcerans*-specific | - |
| MUL_2702 | - | *M. ulcerans*-specific | - |
| MUL_2783 | PE_PGRS39 | *M. ulcerans*-specific | - |
| MUL_2853 | PE_PGRS51 | Rv3367 | PE_PGRS51 |
| MUL_3051 (pseudogene) | - | *M. ulcerans*-specific | - |
| MUL_3056 | PE_PGRS34 | Rv1840c | PE_PGRS34 |
| MUL_3062 | PE_PGRS9_1 | Rv1818c | PE_PGRS33 |
| MUL_3100 (pseudogene) | - | *M. ulcerans*-specific | - |
| MUL_3120 (pseudogene) | - | Rv1768 | PE_PGRS31 |
| MUL_3130 (pseudogene) | - | *M. ulcerans*-specific | - |
| MUL_3164 (pseudogene) | PE_PGRS26 | *M. ulcerans*-specific | - |
| MUL_3186 | - | *M. ulcerans*-specific | - |
| MUL_3209 (pseudogene) | - | *M. ulcerans*-specific | - |
| MUL_3242 (pseudogene) | - | *M. ulcerans*-specific | - |
| MUL_3244 (pseudogene) | - | *M. ulcerans*-specific | - |
| MUL_3247 | PE_PGRS33 | *M. ulcerans*-specific | - |
| MUL_3278 | PE_PGRS46 | Rv2634c | PE_PGRS46 |
| MUL_3292 | - | *M. ulcerans*-specific | - |
| MUL_3318 | - | *M. ulcerans*-specific | - |
| MUL_3342 (pseudogene) | - | *M. ulcerans*-specific | - |
| MUL_3436 (pseudogene) | - | *M. ulcerans*-specific | - |
| MUL_3439 (pseudogene) | - | *M. ulcerans*-specific | - |
| MUL_3485 (pseudogene) | PE_PGRS63 | *M. ulcerans*-specific | - |
| MUL_3507 | - | Rv2162c | PE_PGRS38 |
| MUL_3671 | PE_PGRS2 | *M. ulcerans*-specific | - |
| MUL_3672 | PE_PGRS25 | *M. ulcerans*-specific | - |
| MUL_3704 | PE_PGRS33_1 | *M. ulcerans*-specific | - |
| MUL_3710 (pseudogene) | PE_PGRS7 | *M. ulcerans*-specific | - |
| MUL_3741 | PE_PGRS59 | *M. ulcerans*-specific | - |
| MUL_3851 (pseudogene) | - | *M. ulcerans*-specific | - |
| MUL_3975 | - | *M. ulcerans*-specific | - |
| MUL_3982 | - | *M. ulcerans*-specific | - |
| MUL_3990 | - | *M. ulcerans*-specific | - |
| MUL_4012 (pseudogene) | - | *M. ulcerans*-specific | - |
| MUL_4070 (pseudogene) | PE_PGRS53 | Rv3507 or 8 | PE_PGRS53 or 54 |
| MUL_4086 | - | *M. ulcerans*-specific | - |
| MUL_4087 (pseudogene) | - | *M. ulcerans*-specific | - |
| MUL_4112 (pseudogene) | PE_PGRS27 | *M. ulcerans*-specific | - |
| MUL_4118 (pseudogene) | - | *M. ulcerans*-specific | - |
| MUL_4175 (pseudogene) | - | *M. ulcerans*-specific | - |
| MUL_4177 (pseudogene) | - | Rv3595c | PE_PGRS59 |
| MUL_4212 (pseudogene) | - | *M. ulcerans*-specific | - |
| MUL_4280 | PE_PGRS9_2 | *M. ulcerans*-specific | - |
| MUL_4294 (pseudogene) | PE_PGRS56 | *M. ulcerans*-specific | - |
| MUL_4367 | - | *M. ulcerans*-specific | - |
| MUL_4381 | - | *M. ulcerans*-specific | - |
| MUL_4382 (pseudogene) | - | *M. ulcerans*-specific | - |
| MUL_4399 | - | *M. ulcerans*-specific | - |
| MUL_4400 | - | *M. ulcerans*-specific | - |
| MUL_4419 | - | *M. ulcerans*-specific | - |
| MUL_4420 (pseudogene) | - | *M. ulcerans*-specific | - |
| MUL_4421 (pseudogene) | - | *M. ulcerans*-specific | - |
| MUL_4431 | - | *M. ulcerans*-specific | - |
| MUL_4452 (pseudogene) | - | *M. ulcerans*-specific | - |
| MUL_4489 | - | *M. ulcerans*-specific | - |
| MUL_4524 | - | *M. ulcerans*-specific | - |
| MUL_4525 (pseudogene) | - | *M. ulcerans*-specific | - |
| MUL_4549 (pseudogene) | - | *M. ulcerans*-specific | - |
| MUL_4581 | - | *M. ulcerans*-specific | - |
| MUL_4647 (pseudogene) | - | *M. ulcerans*-specific | - |
| MUL_4718 (pseudogene) | - | *M. ulcerans*-specific | - |
| MUL_4823 (pseudogene) | PE_PGRS1 | Rv0109 | PE_PGRS1 |
| MUL_4871 (pseudogene) | - | *M. ulcerans*-specific | - |
| MUL_4909 (pseudogene) | - | *M. ulcerans*-specific | - |
| MUL_4913 (pseudogene) | - | *M. ulcerans*-specific | - |
| MUL_4981 (pseudogene) | - | *M. ulcerans*-specific | - |
| **PPE genes** | | | |
| ***M. ulcerans* gene number** | ***M. ulcerans* gene name** | ***M. tuberculosis* orthologue gene number** | ***M. tuberculosis* orthologue gene name** |
| MUL_0098 | PPE62 | *M. ulcerans*-specific | - |
| MUL_0134 (pseudogene) | PPE49 | *M. ulcerans*-specific | - |
| MUL_0162 (pseudogene) | - | *M. ulcerans*-specific | - |
| MUL_0399 | - | *M. ulcerans*-specific | - |
| MUL_0492 | - | *M. ulcerans*-specific | - |
| MUL_0597 (pseudogene) | - | *M. ulcerans*-specific | - |
| MUL_0684 (pseudogene) | - | *M. ulcerans*-specific | - |
| MUL_0779 (pseudogene) | - | *M. ulcerans*-specific | - |
| MUL_0782 | - | *M. ulcerans*-specific | - |
| MUL_0786 (pseudogene) | - | *M. ulcerans*-specific | - |
| MUL_0852 (pseudogene) | - | Rv1706c | PPE23 |
| MUL_0890 | - | *M. ulcerans*-specific | - |
| MUL_0892 (pseudogene) | - | *M. ulcerans*-specific | - |
| MUL_0893 | - | *M. ulcerans*-specific | - |
| MUL_0902 | - | *M. ulcerans*-specific | - |
| MUL_0947 | PPE18 | Rv1196 | PPE18 |
| MUL_0964 | - | *M. ulcerans*-specific | - |
| MUL_0965 | - | *M. ulcerans*-specific | - |
| MUL_1154 | - | *M. ulcerans*-specific | - |
| MUL_1202 | PPE3 | Rv0280 | PPE3 |
| MUL_1208 | PPE4 | Rv0286 | PPE4 |
| MUL_1395 | PPE10 | Rv0442c | PPE10 |
| MUL_1470 (pseudogene) | - | *M. ulcerans*-specific | - |
| MUL_1701 | - | *M. ulcerans*-specific | - |
| MUL_1702 | PPE23 | *M. ulcerans*-specific | - |
| MUL_1875 (pseudogene) | - | *M. ulcerans*-specific | - |
| MUL_2094 | - | *M. ulcerans*-specific | - |
| MUL_2096 | - | *M. ulcerans*-specific | - |
| MUL_2098 (pseudogene) | - | *M. ulcerans*-specific | - |
| MUL_2129 | - | *M. ulcerans*-specific | - |
| MUL_2167 | PPE44 | Rv2770c | PPE44 |
| MUL_2169 (pseudogene) | PPE43 | Rv2768c | PPE43 |
| MUL_2203 (pseudogene) | - | *M. ulcerans*-specific | - |
| MUL_2245 | - | *M. ulcerans*-specific | - |
| MUL_2247 | - | *M. ulcerans*-specific | - |
| MUL_2248 (pseudogene) | - | *M. ulcerans*-specific | - |
| MUL_2355 (pseudogene) | PPE37 | Rv2123 | PPE37 |
| MUL_2427 | - | Rv3135 | PPE50 |
| MUL_2428 | PPE51 | Rv3136 | PPE51 |
| MUL_2440 | PPE28 | *M. ulcerans*-specific | - |
| MUL_2456 (pseudogene) | PPE52 | Rv3144c | PPE52 |
| MUL_2473 (pseudogene) | PPE53 | Rv3159c | PPE53 |
| MUL_2584 (pseudogene) | PPE21 | *M. ulcerans*-specific | - |
| MUL_2586 | - | *M. ulcerans*-specific | - |
| MUL_2655 (pseudogene) | PPE14 | *M. ulcerans*-specific | - |
| MUL_2686 | - | *M. ulcerans*-specific | - |
| MUL_2690 | - | *M. ulcerans*-specific | - |
| MUL_2718 | PPE61 | *M. ulcerans*-specific | - |
| MUL_2737 | PPE51_1 | *M. ulcerans*-specific | - |
| MUL_2777 (pseudogene) | PPE5 | *M. ulcerans*-specific | - |
| MUL_2932 | - | *M. ulcerans*-specific | - |
| MUL_2944 (pseudogene) | PPE35 | Rv1918c | PPE35 |
| MUL_3071 | PPE30 | Rv1802 | PPE30 |
| MUL_3072 | PPE32 | Rv1808 | PPE32 |
| MUL_3074 | PPE31 | Rv1807 | PPE31 |
| MUL_3075 (pseudogene) | - | Rv1809 | PPE33 |
| MUL_3076 | PPE31_1 | *M. ulcerans*-specific | - |
| MUL_3087 | PPE26 | Rv1789 | PPE26 |
| MUL_3089 | PPE25 | Rv1787 | PPE25 |
| MUL_3127 | - | *M. ulcerans*-specific | - |
| MUL_3169 | PPE8 | *M. ulcerans*-specific | - |
| MUL_3397 (pseudogene) | PPE6 | *M. ulcerans*-specific | - |
| MUL_3482 (pseudogene) | - | *M. ulcerans*-specific | - |
| MUL_3608 (pseudogene) | - | Rv2356c | PPE40 |
| MUL_3850 (pseudogene) | PPE2 | *M. ulcerans*-specific | - |
| MUL_4121 (pseudogene) | - | *M. ulcerans*-specific | - |
| MUL_4123 (pseudogene) | - | Rv3558 | PPE64 |
| MUL_4195 | PPE65 | Rv3621c | PPE65 |
| MUL_4337 (pseudogene) | PPE46 | *M. ulcerans*-specific | - |
| MUL_4411 (pseudogene) | - | *M. ulcerans*-specific | - |
| MUL_4456 | - | *M. ulcerans*-specific | - |
| MUL_4463 | - | Rv0915c | PPE14 |
| MUL_4491 | - | *M. ulcerans*-specific | - |
| MUL_4521 (pseudogene) | - | *M. ulcerans*-specific | - |
| MUL_4530 (pseudogene) | - | *M. ulcerans*-specific | - |
| MUL_4532 (pseudogene) | - | *M. ulcerans*-specific | - |
| MUL_4621 | PPE15 | Rv1039c | PPE15 |
| MUL_4672 | - | *M. ulcerans*-specific | - |
| MUL_4724 (pseudogene) | - | *M. ulcerans*-specific | - |
| MUL_4853 | PPE1 | Rv0096 | PPE1 |
| MUL_5078 | - | Rv1705c | PPE22 |

* MUL_0946 has been annotated as a PPE in the BuruList database (http://genolist.pasteur.fr/BuruList), given the name PE31 (in contradiction to its annotation as a PPE), but is actually the orthologue of the PE gene Rv1195 (PE13).
