# Supplementary material for: Evolution and expansion of the Mycobacterium tuberculosis PE and PPE multigene families and their association with the duplication of the ESAT-6 (esx) gene cluster regions
Source: BMC Evol Biol. 2006 Nov 15;6:95. doi: 10.1186/1471-2148-6-95 (PMC1660551; doi:10.1186/1471-2148-6-95)
Supplement: Additional file 2 — Comparative genomics for gene size differences between M. tuberculosis H37Rv and CDC1551. The data in this table provide an overview of the reasons for size differences observed between annotated PE and PPE genes from the two M. tuberculosis genome databases, indicating that variation in size due to frameshifts, insertions and deletions is largely associated with the PE_PGRS and PPE-MPTR subfamilies. [file 1471-2148-6-95-S2.doc]

| **Additional file 2** | | | | | | | | | | | | |
| --- | --- | --- | --- | --- | --- | --- | --- | --- | --- | --- | --- | --- |
| **Comparative genomics for gene size differences between M. tuberculosis H37Rv and CDC1551** | | | | | | | | | | | | |
| **PE family (excluding PGRS subfamily)** | | | | | | | | | | | | |
| **Gene name** | **CDC1551 gene no.** | | **CDC1551 gene** **size** | | | H37Rv gene no. | | H37Rv gene size | | Reasons for size difference | *M. bovis* gene no. | |
|
| PE1 | MT0160 | | 1764 | | | Rv0151c | | 1764 | | - | Mb0156c | |
| PE2 | MT0161 | | 1599 | | | Rv0152c | | 1575 | | Alt. start | Mb0157c | |
| PE3 | MT0168 | | 1404 | | | Rv0159c | | 1404 | | - | Mb0164c | |
| PE4 | MT0169 | | 1506 | | | Rv0160c | | 1506 | | - | Mb0165c | |
| PE5 | MT0298 | | 306 | | | Rv0285 | | 306 | | - | Mb0293 | |
| PE6 | MT0349 | | 513 | | | Rv0335c | | 513 | | - | Mb0342c | |
| PE7 | MT0941 | | 297 | | | Rv0916c | | 297 | | - | Mb0940c | |
| PE8 | MT1069 | | 825 | | | Rv1040c | | 825 | | - | Mb1069c | |
| PE9*  PE10 | MT1119  MT1120 | | 450  165 | | | Rv1088  Rv1089 | | 432  360 | | Frameshift | Mb1117  Mb1118 | |
| PE11 | MT1206 | | 318 | | | Rv1169c | | 300 | | Alt. start | Mb1202c | |
| PE12 | MT1209 | | 924 | | | Rv1172c | | 924 | | - | Mb1205c | |
| PE13 | MT1233 | | 333 | | | Rv1195 | | 297 | | Alt. start | Mb1227 | |
| PE14 | MT1252 | | 330 | | | Rv1214c | | 330 | | - | Mb1246c | |
| PE15 | MT1430 | | 306 | | | Rv1386 | | 306 | | - | Mb1421 | |
| PE16 | MT1474 | | 1584 | | | Rv1430 | | 1584 | | - | Mb1465 | |
| PE17 | MT1684 | | 930 | | | Rv1646 | | 930 | | - | Mb1673 | |
| PE18 | MT1837 | | 297 | | | Rv1788 | | 297 | | - | Mb1816 | |
| PE19 | MT1840 | | 315 | | | Rv1791 | | 297 | | Alt. start | Mb1819 | |
| PE20 | MT1855 | | 297 | | | Rv1806 | | 297 | | - | Mb1835 | |
| PE22** | MT2166 | | 294 | | | Rv2107 | | 294 | | - | Mb2131 | |
| PE23 | MT2390 | | 1251 | | | Rv2328 | | 1146 | | Alt. start | Mb2355 | |
| PE24 | MT2481 | | 1002 | | | Rv2408 | | 717 | | Alt. start | Mb2431 | |
| PE25 | MT2506 | | 297 | | | Rv2431c | | 297 | | - | Mb2457c | |
| PE26 | MT2595 | | 1503 | | | Rv2519 | | 1476 | | Alt. start | Mb2548 | |
| PE27 | MT2839 | | 825 | | | Rv2769c | | 825 | | - | Mb2791c | |
| PE27A*** | - | | - | | | Rv3018A | | 84 | |  | Mb3044c | |
| PE29**** | MT3106.1 | | 411 | | | Rv3022A | | 315 | | Alt. start | Mb3048c | |
| PE31***** | MT3581 | | 294 | | | Rv3477 | | 294 | | - | Mb3504 | |
| PE32 | MT3724 | | 297 | | | Rv3622c | | 297 | | - | Deletion | |
| PE33 | MT3752 | | 282 | | | Rv3650 | | 282 | | - | Mb3674 | |
| PE34 | MT3854 | | 369 | | | Rv3746c | | 333 | | Alt. start | Mb3772c | |
| PE35 | MT3986 | | 336 | | | Rv3872 | | 297 | | Alt. start | Mb3902 | |
| PE36 | MT4008 | | 231 | | | Rv3893c | | 231 | | - | Mb3922c | |
| * PE9 and 10 are the result of a frameshift in a gene that most probably originally were part of the PE_PGRS subfamily. This is supported by the fact that these genes are most homologous to the N-terminus of PE_PGRS41 (Rv2396)  ** PE21 is not included in this table. A frameshift mutation gave rise to PE21 (Rv2099c) and PE_PGRS36 (Rv2098c) in *M. tuberculosis* H37Rv. PE21 is thus actually part of the PGRS gene PE_PGRS36 and can be found in the PGRS subfamily table. This has been authenticated by the presence of a single intact orthologue of this ancestral gene in the genomes of both *M. tuberculosis* CDC1551 (namely MT2159) and *M. bovis* (namely Mb2125c).  *** PE27A was identified after the original annotation of the genome of *M. tuberculosis* H37Rv  **** PE28 (MT3105 - Rv3020c) and MT0300 (Rv0287 or TB9.8) was annotated incorrectly as PE family proteins in the *M. tuberculosis* H37Rv and CDC 1551 genome annotations. These are two CFP-10 proteins.  ***** PE30 has been re-annotated as PE_PGRS63 in the H37Rv database, see PGRS subfamily table. | | | | | | | | | | | | |
| **Additional file 2 *(continued)*** | | | | | | | | | | | | |
| **PE_PGRS subfamily** | | | | | | | | | | | | |
| **Gene name** | | **CDC1551 gene no.** | | **CDC1551 gene size** | **H37Rv gene no.** | | **H37Rv gene size** | | **Reasons for size difference** | | | **M. bovis gene no.** |
|
| PE_PGRS1 | | MT0118 | | 1599 | Rv0109 | | 1488 | | Alt. start | | | Mb0113 |
| PE_PGRS2 | | MT0132 | | 1683 | Rv0124 | | 1461 | | Alt. start and Insertion in repeat region | | | Mb0129 |
| PE_PGRS3 | | MT0291 | | 2715 | Rv0278c | | 2871 | | Alt. start and Insertion in N- terminus | | | Mb0284c  Mb0285c  Mb0286c |
| PE_PGRS4 | | - | | - | Rv0279c | | 2511 | | Not annotated in CDC database (rev. compl. of MT0291.4) frameshift | | | Mb0287c |
| PE_PGRS5 | | MT0311 | | 1866 | Rv0297 | | 1773 | | Alt. start | | | Mb0305 |
| PE_PGRS6 | | MT0556 | | 1866 | Rv0532 | | 1782 | | Insertion | | | Mb0545  Mb0546 |
| PE_PGRS7 | | MT0607 | | 3918 | Rv0578c | | 3918 | | - | | | Mb0593c |
| PE_PGRS8 | | MT0768 | | 516 | Rv0742 | | 525 | | Alt. start | | | Mb0763 |
| PE_PGRS9 | | MT0772.1 | | 2349 | Rv0746 | | 2349 | | - | | | Mb0767 |
| PE_PGRS10 | | MT0772.5 | | 2355 | Rv0747 | | 2403 | | InDel | | | Mb0768 |
| PE_PGRS11 | | MT0778 | | 1752 | Rv0754 | | 1752 | | - | | | Mb0776 |
| PE_PGRS12  PE_PGRS13 | | MT0854  MT0854.1 | | 450  2247 | Rv0832  Rv0833 | | 411  2247 | | Frameshift | | | Mb0855  Mb0856 |
| PE_PGRS14 | | MT0855 | | 2637 | Rv0834c | | 2646 | | InDel | | | Mb0857c |
| PE_PGRS15 | | MT0894 | | 1827 | Rv0872c | | 1818 | | InDel | | | Mb0896c |
| PE_PGRS16 | | MT1004 | | 2801 | Rv0977 | | 2769 | | Alt. start Frameshift | | | Mb1002 |
| PE_PGRS17 | | MT1006.1 | | 1005 | Rv0978c | | 993 | | InDel | | | Mb1003c |
| PE_PGRS18 | | MT1008 | | 1428 | Rv0980c | | 1371 | | Alt. start, InDel | | | Mb1006c |
| PE_PGRS19 | | MT1096.1 | | 2001 | Rv1067c | | 2001 | | - | | | Mb1096c |
| PE_PGRS20 | | MT1097 | | 1599 | Rv1068c | | 1389 | | Alt. start, InDel | | | Mb1097c |
| PE_PGRS21 | | MT1118.1 | | 2306 | Rv1087 | | 2301 | | InDels, Frameshift | | | Mb1116 |
| PE_PGRS22 | | MT1123 | | 1905 | Rv1091 | | 2559 | | InDel | | | Mb1121 |
| PE_PGRS23 | | MT1280.1 | | 1761 | Rv1243c | | 1689 | | Alt. start | | | Mb1275c |
| PE_PGRS24 | | MT1367 | | 1608 | Rv1325c | | 1812 | | Alt. start | | | Mb1360c |
| PE_PGRS25 | | MT1440.1 | | 1727 | Rv1396c | | 1728 | | Frameshift | | | Mb1431c |
| PE_PGRS26 | | MT1486 | | 1472 | Rv1441c | | 1473 | | Frameshift | | | Mb1476c |
| PE_PGRS27 | | MT1497.1 | | 4224 | Rv1450c | | 3987 | | Alt. start, InDels | | | Mb1485c |
| PE_PGRS28 | | MT1499 | | 2214 | Rv1452c | | 2223 | | Alt. start, InDels | | | Mb1487c |
| PE_PGRS29 | | MT1514.1 | | 1125 | Rv1468c | | 1110 | | Alt. start | | | Mb1503c |
| PE_PGRS30 | | MT1689 | | 3033 | Rv1651c | | 3033 | | - | | | Mb1679c |
| wag22 | | MT1807 | | 2741 | Rv1759c | | 2742 | | Frameshift (aa pos. 85) | | | Mb1789c  Mb1790c |
| PE_PGRS31 | | MT1818 | | 1852 | Rv1768 | | 1854 | | Frameshift (aa pos. 392) | | | Mb1797 |
| PE_PGRS32 | | MT1853 | | 1950 | Rv1803c | | 1917 | | Alt. start | | | Mb1831c  Mb1832c |
| PE_PGRS33 | | MT1866 | | 1473 | Rv1818c | | 1494 | | Alt. start, InDel | | | Mb1849c |
| PE_PGRS34 | | MT1888 | | 1545 | Rv1840c | | 1545 | | - | | | Mb1871c |
| PE_PGRS35 | | MT2036 | | 1674 | Rv1983 | | 1674 | | - | | | Mb2005 |
| PE21*  PE_PGRS36 | | MT2159 | | 1473 | Rv2099c  Rv2098c | | 174  1302 | | Frameshift | | | Mb2125c |
| PE_PGRS37 | | MT2185.1 | | 1148 | Rv2126c | | 768 | | Alt. start, Frameshift, Pseudogene (stopcodons at aa pos 11, 117, 160 and 237) | | | Mb2150c |
| PE_PGRS38 | | MT2220 | | 1596 | Rv2162c | | 1596 | | - | | | Mb2186c |
| PE_PGRS39 | | MT2404 | | 1239 | Rv2340c | | 1239 | | - | | | Mb2369c |
| PE_PGRS40 | | MT2440 | | 285 | Rv2371 | | 183 | | Alt. start | | | Mb2392 |
| PE_PGRS41 | | MT2467.1 | | 1146 | Rv2396 | | 1083 | | Alt. start | | | Mb2418 |
| PE_PGRS42 | | MT2561 | | 2082 | Rv2487c | | 2082 | | - | | | Mb2512c  Mb2513c  Mb2514c |
| PE_PGRS43 | | MT2564 | | 4995 | Rv2490c | | 4980 | | Alt. start | | | Mb2517c  Mb2518c |
| PE_PGRS44 | | MT2668.1 | | 1515 | Rv2591 | | 1629 | | Alt. start | | | Mb2622 |
| PE_PGRS45 | | MT2690 | | 1428 | Rv2615c | | 1383 | | Alt. start | | | Mb2648c |
| PE_PGRS46 | | MT2712 | | 2364 | Rv2634c | | 2334 | | Alt. start | | | Mb2667c |
| PE_PGRS47 | | MT2812 | | 1362 | Rv2741 | | 1575 | | InDel | | | Mb2761 |
| PE_PGRS48 | | MT2919 | | 1989 | Rv2853 | | 1845 | | Alt. start | | | Mb2878 |
| PE_PGRS63** | | MT3181 | | 1311 | Rv3097c | | 1311 | | - | | | Mb3124c |
| PE_PGRS49  PE_PGRS50 | | MT3448  MT3449 | | 1014  4521 | Rv3344c  Rv3345c | | 1452  4614 | | Alt.start, InDel, Frameshift | | | Mb3376c  Mb3377c |
| PE_PGRS51 | | MT3476 | | 1884 | Rv3367 | | 1764 | | Alt. start, InDel | | | Mb3402 |
| PE_PGRS52 | | MT3495 | | 2325 | Rv3388 | | 2193 | | Alt. start | | | Mb3420 |
| PE_PGRS53 | | MT3612 | | 4152 | Rv3507 | | 4143 | | InDel | | | Mb3537 |
| PE_PGRS54 | | MT3612.1 | | 5145 | Rv3508 | | 5703 | | Alt. start, InDels | | | Mb3538 |
| PE_PGRS55  PE_PGRS56 | | MT3615.1 | | 5697 | Rv3511  Rv3512 | | 2142  3237 | | Frameshift | | | Mb3541 |
| PE_PGRS57 | | MT3615.3 | | 3651 | Rv3514 | | 4467 | | InDels | | | Mb3543 |
| PE_PGRS58 | | MT3696 | | 1752 | Rv3590c | | 1752 | | - | | | Mb3621c |
| PE_PGRS59 | | MT3701 | | 1308 | Rv3595c | | 1317 | | InDel | | | Mb3626c |
| PE_PGRS60  PE_PGRS61 | | Not annotated  MT3756 | | -  744 | Rv3652  Rv3653 | | 159  735 | | Original split caused by frameshift, Alt. start, InDel | | | Mb3676  Mb3677 |
| PE_PGRS62 | | MT3920 | | 1512 | Rv3812 | | 1512 | | - | | | Mb3842 |

* A frameshift mutation gave rise to PE21 (Rv2099c) and PE_PGRS36 (Rv2098c) in *M. tuberculosis* H37Rv. PE21 is thus actually part of the PGRS gene PE_PGRS36. This has been authenticated by the presence of a single intact orthologue of this ancestral gene in the genomes of both *M. tuberculosis* CDC1551 (namely MT2159) and *M. bovis* (namely Mb2125c).

** PE_PGRS63 was identified after the initial annotation and is situated between PE_PGRS48 and PE_PGRS49.

| **Additional file 2 *(continued)*** | | | | | | |
| --- | --- | --- | --- | --- | --- | --- |
| **PPE family (including MPTR subfamily*)** | | | | | | |
| **Gene name** | **CDC1551 gene no.** | **CDC1551 gene size** | **H37Rv gene no.** | **H37Rv gene size** | **Reasons for size difference** | **M. bovis gene no.** |
|
| PPE1 | MT0105 | 1389 | Rv0096 | 1389 | - | Mb0099 |
| PPE2 | MT0269 | 1668 | Rv0256c | 1668 | - | Mb0262c |
| PPE3 | MT0292 | 1725 | Rv0280 | 1608 | Alt. start | Mb0288 |
| PPE4 | MT0299 | 1539 | Rv0286 | 1539 | - | Mb0294 |
| ***PPE5***  ***PPE6*** | MT0318 | 9558 | Rv0304c  Rv0305c | 6612  2889 | Frameshift | Mb0312c  Mb0313c |
| ***PPE7***  ***PPE8*** | MT0369 MT0370 | 555  9825 | Rv0354c  Rv0355c | 423  9900 | Frameshift | Mb0362c |
| -  PPE9 | MT0400 | 1329 | Rv0387c  Rv0388c | 732  540 | Frameshift | Mb0394c |
| ***PPE10*** | MT0458 | 1461 | Rv0442c | 1461 | - | Mb0450c |
| PPE11 | MT0469 | 1554 | Rv0453 | 1554 | - | Mb0461 |
| ***PPE12*** | MT0779 | 1938 | Rv0755c | 1935 | Alt. start | Mb0777c |
| ***PPE13*** | MT0901 | 1332 | Rv0878c | 1329 | Alt. start | Mb0902c |
| PPE14 | MT0940 | 1278 | Rv0915c | 1269 | Alt. start | Mb0939c |
| PPE15 | MT1068 | 1173 | Rv1039c | 1173 | - | Mb1068c |
| ***PPE16*** | MT1168 | 1908 | Rv1135c | 1854 | Alt. start | Mb1166c |
| PPE17 | MT1205 | 1038 | Rv1168c | 1038 | - | Mb1200c  Mb1201c |
| PPE18 | MT1234 | 1173 | Rv1196 | 1173 | - | Mb1228 |
| PPE19 | MT1406 | 1188 | Rv1361c | 1188 | - | Mb1396c |
| PPE20 | MT1431 | 1617 | Rv1387 | 1617 | - | Mb1422 |
| ***PPE21*** | MT1599 | 2280 | Rv1548c | 2034 | Alt. start | Mb1575c |
| PPE22 | MT1745 | 1155 | Rv1705c | 1155 | - | Mb1731c |
| PPE23 | MT1746 | 1182 | Rv1706c | 1182 | - | Mb1732c |
| ***PPE24*** | MT1796 | 3315 | Rv1753c | 3159 | Insert in repeat region | Mb1782c |
| PPE25 | MT1836 | 1215 | Rv1787 | 1095 | Alt. start | Mb1815 |
| PPE26 | MT1838 | 1233 | Rv1789 | 1179 | Alt. start | Mb1817 |
| PPE27 | MT1839 | 1089 | Rv1790 | 1050 | Alt. start | Mb1818 |
| ***PPE28*** | MT1849 | 1965 | Rv1800 | 1965 | - | Mb1828 |
| PPE29 | MT1850 | 1269 | Rv1801 | 1269 | - | Mb1829 |
| PPE30 | MT1851 | 1389 | Rv1802 | 1389 | - | Mb1830 |
| PPE31 | MT1856 | 1209 | Rv1807 | 1197 | Alt. start | Mb1836 |
| PPE32 | MT1856.1 | 1158 | Rv1808 | 1227 | Alt. start | Mb1837 |
| PPE33 | MT1857 | 2082 | Rv1809 | 1404 | Alt. start | Mb1838  Mb1839 |
| ***PPE34*** | MT1968 | 4473 | Rv1917c | 4377 | Alt. start, indels in repeat region | Mb1951c |
| ***PPE35*** | MT1969 | 3039 | Rv1918c | 2961 | Alt. start | Mb1952c  Mb1953c |
| PPE36 | MT2167 | 729 | Rv2108 | 729 | - | Mb2132 |
| PPE37 | MT2182.1 | 450 | Rv2123 | 1419 | 969 bp of C-terminus + 1196 bp of *metH* C-terminus deleted | Mb2147 |
| PPE38 | MT2419 | 1173 | Rv2352c | 1173 | - | Deleted (RD5) |
| PPE71** | MT2422 | 1245 | - | - | Copy of MT2419 | - |
| ***PPE39*** | MT2423 | 1863 | Rv2353c | 1062 | N-terminus disrupted by IS*6110* insertion | Mb2376c (1374bp,C-term. deleted by RD5) |
| ***PPE40*** | MT2425 | 1845 | Rv2356c | 1845 | - | Mb2377c |
| PPE41 | MT2505 | 669 | Rv2430c | 582 | Alt. start | Mb2456c |
| ***PPE42*** | MT2683 | 1740 | Rv2608 | 1740 | - | Mb2640 |
| PPE43 | MT2838 | 1386 | Rv2768c | 1182 | Alt. start | Mb2790c |
| PPE44 | MT2840 | 1206 | Rv2770c | 1146 | Alt. start | Mb2792c |
| PPE45 | MT2959 | 1224 | Rv2892c | 1224 | - | Mb2916c |
| PPE46 | MT3098  MT3101 | 405  930 | Rv3018c | 1302 | Gene split by IS*6110* insertion | Mb3043c |
| PPE47  PPE48 | MT3106 | 1305 | Rv3021c  Rv3022c | 1074  243 | Frameshift | Mb3047c |
| PPE49 | MT3209 | 1173 | Rv3125c | 1173 | - | Mb3148c |
| PPE50 | - | - | Rv3135 | 396 | Copy of Rv3136 | Mb3159 |
| PPE51 | MT3221 | 1140 | Rv3136 | 1140 | - | Mb3160 |
| ***PPE52*** | MT3231 | 1227 | Rv3144c | 1227 | - | Mb3168c |
| ***PPE53*** | MT3247 | 1809 | Rv3159c | 1770 | Alt. start | Mb3183c |
| ***PPE70***** | MT3248 | 2058 | - | - | Copy of MT3247 | Mb3184c |
| ***PPE54*** | MT3447 | 2285 | Rv3343c | 7569 | Indel in repeat region, frameshift | Mb3375c |
| ***PPE55*** | MT3453 | 9498 | Rv3347c | 9471 | Alt. stop due to frameshift | Mb3379c  Mb3380c |
| ***PPE56*** | MT3458 | 11353 | Rv3350c | 11148 | Alt. start | Mb3383c  Mb3384c  Mb3385c |
| PPE57 | - | - | Rv3425 | 531 | Duplicate of Rv3429 | Mb3459 (537bp, divergent C-term.) |
| PPE58 | - | - | Rv3426 | 696 | Duplicate of Rv3429 | Deletion (RD6) |
| PPE59 | MT3533 | 534 | Rv3429 | 534 | - | Deletion (RD6) |
| PPE60 | MT3582 | 1179 | Rv3478 | 1179 | - | Mb3505 |
| PPE61 | MT3636 | 1218 | Rv3532 | 1218 | - | Mb3562 |
| ***PPE62*** | MT3637 | 1746 | Rv3533c | 1746 | - | Mb3563c |
| PPE63*** | MT3643 | 1437 | Rv3539 | 1437 | - | Mb3569 |
| ***PPE64*** | MT3663 | 1869 | Rv3558 | 1656 | Alt. stop due to frameshift | Mb3588 |
| PPE65 | MT3723 | 1239 | Rv3621c | 1239 | - | Deleted |
| PPE66  PPE67 | MT3844 | 1230 | Rv3738c  Rv3739c | 945  231 | Point mutation | Mb3765c (357bp, N-term. deleted with ortholog of PPE67) |
| PPE68 | MT3987 | 1113 | Rv3873 | 1104 | Alt. start | Mb3903 |
| PPE69 | MT4007 | 1197 | Rv3892c | 1197 | - | Mb3921c |
| *Members of the MPTR subfamily are indicated in italics and bold  ** PPE70 and PPE71 is not present in *M. tuberculosis* H37Rv and was identified after the initial annotation of H37Rv in CDC1551. These genes are situated between PPE53 and PPE54, and PPE38 and PPE39, respectively.  ***PPE63 (Rv3539) was originally incorrectly annotated as a member of the PE family | | | | | | |
