# Supplementary material for: Evolution and expansion of the Mycobacterium tuberculosis PE and PPE multigene families and their association with the duplication of the ESAT-6 (esx) gene cluster regions
Source: BMC Evol Biol. 2006 Nov 15;6:95. doi: 10.1186/1471-2148-6-95 (PMC1660551; doi:10.1186/1471-2148-6-95)
Supplement: Additional file 3 — Comparative genomics for extent of sequence variation between M. tuberculosis H37Rv and CDC1551. The data in this table provide an overview of the extent of sequence variation on a protein level between the orthologues of the PE and PPE families in the two M. tuberculosis strains, indicating that the "ancestral-type" PE and PPE genes are highly conserved between strains, while the PPE-MPTR and PE_PGRS subfamilies are prone to sequence variation. [file 1471-2148-6-95-S3.doc]

| **Additional file 3** | | | | | |
| --- | --- | --- | --- | --- | --- |
| **Comparative genomics for extent of sequence variation between M. *tuberculosis* H37Rv and CDC1551** | | | | | |
| **PE family (excluding PGRS subfamily)** | | | | | |
| **Gene name** | **Number of Protein Mismatches (aa position, change)*** | **Number of Protein Conservative Substitutions (aa position, change)*** | **Number of Indels (aa position, no of DNA bp)*** | **Protein percentages** | |
|  |  |  |  | **Identity** | **Similarity** |
| PE1 | 1 (122, T-A) | 1 (572, V-I) | - | 99.7% | 99.8% |
| PE2 | - | - | - | 100% | 100% |
| PE3 | 2 (14, A-T, 368, W-R) | - | - | 99.6% | 99.6% |
| PE4 | - | - | - | 100% | 100% |
| PE5 | - | - | - | 100% | 100% |
| PE6 | - | - | - | 100% | 100% |
| PE7 | - | - | - | 100% | 100% |
| PE8 | - | - | - | 100% | 100% |
| PE9**  PE10 | - | - | - | 100%  100% | 100%  100% |
| PE11 | - | - | - | 100% | 100% |
| PE12 | - | - | - | 100% | 100% |
| PE13 | - | - | - | 100% | 100% |
| PE14 | - | - | - | 100% | 100% |
| PE15 | - | - | - | 100% | 100% |
| PE16 | - | - | - | 100% | 100% |
| PE17 | - | - | - | 100% | 100% |
| PE18 | - | - | - | 100% | 100% |
| PE19 | - | - | - | 100% | 100% |
| PE20 | - | - | - | 100% | 100% |
| PE22*** | - | - | - | 100% | 100% |
| PE23 | - | - | - | 100% | 100% |
| PE24 | - | - | - | 100% | 100% |
| PE25 | - | - | - | 100% | 100% |
| PE26 | - | - | - | 100% | 100% |
| PE27 | 1 (51, R-L) | 1 (270, M-V) | - | 99.3% | 99.6% |
| PE27A**** | - | - | - | - | - |
| PE29***** | - | - | - | 100% | 100% |
| PE31****** | - | - | - | 100% | 100% |
| PE32 | - | - | - | 100% | 100% |
| PE33 | - | - | - | 100% | 100% |
| PE34 | - | - | - | 100% | 100% |
| PE35 | - | - | - | 100% | 100% |
| PE36 | - | - | - | 100% | 100% |

* Change from CDC1551 to H37Rv

** PE9 and 10 are the result of a frameshift in a gene that most probably originally were part of the PE_PGRS subfamily. This is supported by the fact that these genes are most homologous to the N-terminus of PE_PGRS41 (Rv2396)

*** PE21 is not included in this table. A frameshift mutation gave rise to PE21 (Rv2099c) and PE_PGRS36 (Rv2098c) in *M. tuberculosis* H37Rv. PE21 is thus actually part of the PGRS gene PE_PGRS36 and can be found in the PGRS subfamily table. This has been authenticated by the presence of a single intact orthologue of this ancestral gene in the genomes of both *M. tuberculosis* CDC1551 (namely MT2159) and *M. bovis* (namely Mb2125c).

**** PE27A was identified after the original annotation of the genome of *M. tuberculosis* H37Rv

***** PE28 (MT3105 - Rv3020c) and MT0300 (Rv0287 or TB9.8) was annotated incorrectly as PE family proteins in the *M. tuberculosis* H37Rv and CDC 1551 genome annotations. These are two CFP-10 proteins.

****** PE30 has been re-annotated as PE_PGRS63 in the H37Rv database, see PGRS subfamily table.

| **Additional file 3 *(continued)*** | | | | | |
| --- | --- | --- | --- | --- | --- |
| **PE_PGRS subfamily** | | | | | |
| **Gene name** | **Number of Protein Mismatches (aa position, change)*** | **Number of Protein Conservative Substitutions (aa position, change)*** | Number of Indels (aa position, no of DNA bp)* | **Protein Percentages** | |
|  |  |  |  | **Identity** | **Similarity** |
| PE_PGRS1 | 1 (346, G-R) | - | - | 99.8% | 99.8% |
| PE_PGRS2 | 3 (266, G-I, 268, G-L, 269, A-G) | - | 1 (455, 180 bp) | 99.0% | 99.0% |
| PE_PGRS3 | 1 (807, G-R) | - | 1 (162, 18 bp) | 99.7% | 99.8% |
| PE_PGRS4 | 4 (95, T-V, 325, N-S, 352, C-G, 372, I-S) frameshift | - | 1 (410, 33 bp) | - | - |
| PE_PGRS5 | 2 (158, T-A, 445, V-A) | - | - | 99.7% | 99.7% |
| PE_PGRS6 | 3 (227, G-D, 239, G-A, 380, V-G) | - | 1 (169, 84 bp) | 99.3% | 99.3% |
| PE_PGRS7 | 1 (363, R-G) | - | - | 99.9% | 99.9% |
| PE_PGRS8 | - | - | - | 100% | 100% |
| PE_PGRS9 | 4 (191, G-E, 252, A-T, 320, A-T, 445, A-T) | 2 (263, M-L, 280, D-N) | - | 99.2% | 99.7% |
| PE_PGRS10 | 6 (188, S-G, 225, G-R, 227, G-R, 300, G-S, 338, I-T, 377, P-A) | 1 (295, R-K) | 1 (577, 48 bp) | 98.9% | 99.0% |
| PE_PGRS11 | 1 (497, L-P) | - | - | 99.8% | 99.8% |
| PE_PGRS12  PE_PGRS13 | Frameshift | Frameshift | Frameshift | 100%  99.7% | 100%  99.7% |
| PE_PGRS14 | 1 (804, G-N) | - | 1 (773, 9 bp) | 99.8% | 99.8% |
| PE_PGRS15 | - | - | 1 (606, 9 bp) | 100% | 100% |
| PE_PGRS16 | Frameshift | Frameshift | Frameshift | - | - |
| PE_PGRS17 | 11 (99, Q-N, 102, G-D, 103, V-A, 106, T-A, 108, T-V, 112, V-T, 115, K-P, 124, A-I, 125, P-D 133, A-N, 136, I-W) | 4 (100, I-V, 109, E-Q, 110, A-S, 122, H-N) | 1 (101, 12 bp) | 95.2% | 96.4% |
| PE_PGRS18 | 13 (99, Q-N, 102, G-D, 103, V-A, 106, T-A, 108, T-V, 112, V-T, 115, K-P, 120, R-G, 124, A-I, 125, P-D, 133, A-N, 136, I-W 170, T-A) | 4 (100, I-V, 109, E-Q, 110, A-S, 122, H-N) | 1 (101, 12 bp) | 96.1% | 96.9% |
| PE_PGRS19 | 1 (111, W-L) | - | - | 99.9% | 99.9% |
| PE_PGRS20 | - | 1 (224, S-T) | 1 (242, 192 bp) | 99.6% | 99.8% |
| PE_PGRS21 | - Frameshift | - Frameshift | 2 (189, 78 bp, 712, 45 bp) Frameshift | - | - |
| PE_PGRS22 | - | - | 1 (350, 654 bp) | 99.8% | 99.8% |
| PE_PGRS23 | - | - | - | 100% | 100% |
| PE_PGRS24 | 3 (132, V-G, 135, D-G, 337, D-G) | 1 (508, F-L) | - | 99.3% | 99.5% |
| PE_PGRS25 | 1 (66, S-R) Frameshift | - Frameshift | - Frameshift | - | - |
| PE_PGRS26 | 1 (229, D-G) Frameshift | - Frameshift | - Frameshift | - | - |
| PE_PGRS27 | 1 (631, S-G) | - | 2 (367, 207 bp, 704, 27 bp) | 99.5% | 99.5% |
| PE_PGRS28 | 16 (323, A-T, 339, D-A, 345, A-T, 351, G-A, 353, H-F, 363, T-N, 365, Q-G, 366, A-D, 369, A-N, 372, S-M, 378, V-A, 380, V-P, 416, A-G, 417, N-P, 429, H-A, 438, T-A) | 5 (342, I-V, 358, V-I, 359, I-V, 432, N-H, 435, N-D) | 2 (355, 6 bp, 361, 6 bp) | 96.9% | 97.6% |
| PE_PGRS29 | - | - | - | 100% | 100% |
| PE_PGRS30 | - | 1 (627, L-F) | - | 99.9% | 100% |
| wag22 | - Frameshift | - Frameshift | - Frameshift | 99.9% | 99.9% |
| PE_PGRS31 | - Frameshift | - Frameshift | - Frameshift | - | - |
| PE_PGRS32 | 9 (517, C-G, 519, S-G, 539, S-G, 545, S-G, 548, R-G, 554, S-G, 557, S-G, 572, R-G, 574, L-P, | - | - | 98.1% | 98.1% |
| PE_PGRS33 | - | - | 2 (191, 90 bp, 415, 9 bp) | 99.6% | 99.6% |
| PE_PGRS34 | - | - | - | 100% | 100% |
| PE_PGRS35 | - | - | - | 100% | 100% |
| PE21**  PE_PGRS36 | Frameshift | Frameshift | - | 100%  99.5% | 100%  99.5% |
| PE_PGRS37 | 1 (193, S-N) | - | - | - | - |
| PE_PGRS38 | - | - | - | 100% | 100% |
| PE_PGRS39 | - | - | - | 100% | 100% |
| PE_PGRS40 | - | - | - | 100% | 100% |
| PE_PGRS41 | - | - | - | 100% | 100% |
| PE_PGRS42 | - | - | - | 100% | 100% |
| PE_PGRS43 | - | - | - | 100% | 100% |
| PE_PGRS44 | - | - | - | 100% | 100% |
| PE_PGRS45 | - | - | - | 100% | 100% |
| PE_PGRS46 | 1 (274, T-A) | 2 (51, L-V, 63, H-Q) | - | 99.6% | 99.9% |
| PE_PGRS47 | - | - | 1 (270, 213 bp) | 99.8% | 99.8% |
| PE_PGRS48 | 1 (180, G-R) | - | - | 99.5% | 99.5% |
| PE_PGRS63*** | 1 (427, L-P) | - | - | 99.8% | 99.8% |
| PE_PGRS49  PE_PGRS50 | - 2 (800, T-N, 870, R-G)  Frameshift | - 2 (1364, H-N, 1367, N-H) Frameshift | - 3 (502, 78 bp, 966, 189 bp, 1420, 18 bp) Frameshift | 100%  99.5% | 100%  99.7% |
| PE_PGRS51 | - | - | 1 (378, 9 bp) | 99.8% | 99.8% |
| PE_PGRS52 | 1 (503, R-H) | - | - | 99.7% | 99.7% |
| PE_PGRS53 | - | - | 4 (557, 9 bp, 745, 18 bp, 845, 9 bp, 1305, 9 bp) | 99.7% | 99.7% |
| PE_PGRS54 | 33 (195, S-G, 1470, APA-QNG, 1478, LI-SE, 1482, PD-AG, 1649, A-V, 1696, D-Q, 1702, E-A, 1705, G-N, 1713, A-P, 1715, AA-DG, 1718, A-I, 1721, N-T, 1724, K-A, 1727, D-A, 1730, T-A, 1732, GT-AD, 1735, GT-DP, 1738, FA-ID, 1748, R-H, 1751, A-Q, 1754, N-K, 1769, V-D, 1771, D-G, 1773, A-G, 1778, D-A) | 5 (1180, A-S, 1232, N-S, 1710, S-A, 1737, N-S, 1767, N-S) | 7 (598, 75 bp, 842, 612 bp, 1474, 12 bp, 1485, 6 bp, 1699, 9 bp, 1756, 30 bp, 1774, 3 bp) | 97.3% | 97.6% |
| PE_PGRS55  PE_PGRS56 | Frameshift | Frameshift | Frameshift | 99.6% | 99.7% |
| PE_PGRS57 | 15 (335, M-T, 341, A-D, 345, NPT-VLG, 353, D-K, 356, T-V, 359, S-V, 362, E-L, 732, A-V, 754, G-D, 857, G-C, 871, G-C, 874, GG-VV, | 3 (347, I-V, 738, N-S, 852, A-S) | 8 (343, 6 bp, 442, 36 bp, 562, 75 bp, 654, 228 bp, 855, 9 bp, 952, 9 bp, 1046, 606 bp, 1346, 117) | 97.8% | 98.1% |
| PE_PGRS58 | - | - | - | 100% | 100% |
| PE_PGRS59 | - | - | 1 (373, 9 bp) | 99.8% | 99.8% |
| PE_PGRS60  PE_PGRS61 | - | - | - 1 (189, 18 bp) | 100%  99.6% | 100%  99.6% |
| PE_PGRS62 | - | - | - | 100% | 100% |

* Change from CDC1551 to H37Rv

** A frameshift mutation gave rise to PE21 (Rv2099c) and PE_PGRS36 (Rv2098c) in *M. tuberculosis* H37Rv. PE21 is thus actually part of the PGRS gene PE_PGRS36. This has been authenticated by the presence of a single intact orthologue of this ancestral gene in the genomes of both *M. tuberculosis* CDC1551 (namely MT2159) and *M. bovis* (namely Mb2125c).

*** PE_PGRS63 was identified after the initial annotation and is situated between PE_PGRS48 and PE_PGRS49.

| **Additional file 3 *(continued)*** | | | | | |
| --- | --- | --- | --- | --- | --- |
| **PPE family (excluding MPTR subfamily)** | | | | | |
| **Gene name** | **Number of Protein Mismatches (aa position, change)*** | **Number of Protein Conservative Substitutions (aa position, change)*** | Number of Indels (aa position, no of DNA bp)* | **Protein Percentages** | |
|  |  |  |  | **Identity** | **Similarity** |
| PPE1 | - | - | - | 100% | 100% |
| PPE2 | 1 (404, L-P) | 1 (461, S-A) | - | 99.6% | 99.8% |
| PPE3 | 1 (337, P-S) | - | - | 99.8% | 99.8% |
| PPE4 | - | - | - | 100% | 100% |
| -  PPE9 | Frameshift | Frameshift | Frameshift | 100%  93.9% | 100%  96.7% |
| PPE11 | 1 (430, C-R) | - | - | 99.8% | 99.8% |
| PPE14 | - | - | - | 100% | 100% |
| PPE15 | - | - | - | 100% | 100% |
| PPE17 | - | - | - | 100% | 100% |
| PPE18 | - | - | - | 100% | 100% |
| PPE19 | 2 (158, A-T, 159, T-A) | - | - | 99.5% | 99.5% |
| PPE20 | - | - | - | 100% | 100% |
| PPE22 | 1 (364, T-M) | - | - | 99.7% | 99.7% |
| PPE23 | - | - | - | 100% | 100% |
| PPE25 | 1 (283, V-A) | - | - | 99.7% | 99.7% |
| PPE26 | - | - | - | 100% | 100% |
| PPE27 | - | - | - | 100% | 100% |
| PPE29 | - | - | - | 100% | 100% |
| PPE30 | 1 (401, S-L) | - | - | 99.8% | 99.8% |
| PPE31 | 1 (227, S-F) | 1 (238, L-V) | - | 99.5% | 99.8% |
| PPE32 | - | - | - | 100% | 100% |
| PPE33 | - | 1 (252, L-V) | - | 99.8% | 100% |
| PPE36 | - | - | - | 100% | 100% |
| PPE37 | Deletion | Deletion | Deletion | 68.3% | 77.2% |
| PPE38 | - | - | - | 100% | 100% |
| PPE71** | - | - | - | - | - |
| PPE41 | - | - | - | 100% | 100% |
| PPE43 | - | - | - | 100% | 100% |
| PPE44 | - | - | - | 100% | 100% |
| PPE45 | - | - | - | 100% | 100% |
| PPE46 | IS*6110* insertion | IS*6110* insertion | IS*6110* insertion | 99.2%  100% | 100%  100% |
| PPE47  PPE48 | Frameshift | Frameshift | Frameshift | 98.6%  98.9% | 99.4%  99.4% |
| PPE49 | - | - | - | 100% | 100% |
| PPE50 | - | - | - | - | - |
| PPE51 | - | - | - | 100% | 100% |
| PPE57 | - | - | - | - | - |
| PPE58 | - | - | - | - | - |
| PPE59 | - | - | - | 100% | 100% |
| PPE60 | 29 | 15 | - | 88,8% | 92.6% |
| PPE61 | - | - | - | - | - |
| PPE63*** | - | - | - | 100% | 100% |
| PPE65 | - | - | - | 100% | 100% |
| PPE66  PPE67 | Point mutation | Point mutation | Point mutation | - | - |
| PPE68 | - | - | - | 100% | 100% |
| PPE69 | 3 (19, K-T, 114, V-E, 204, C-D) | - | - | 99.2% | 99.2% |

* Change from CDC1551 to H37Rv

** PPE71 is not present in *M. tuberculosis* H37Rv and was identified after the initial annotation of H37Rv in CDC1551. This gene is situated between PPE38 and PPE39.

***PPE63 (Rv3539) was originally incorrectly annotated as a member of the PE family

| **Additional file 3 *(continued)*** | | | | | |
| --- | --- | --- | --- | --- | --- |
| **PPE-MPTR subfamily** | | | | | |
| **Gene name** | **Number of Protein Mismatches (aa position, change)*** | **Number of Protein Conservative Substitutions (aa position, change)*** | Number of Indels (aa position, no of DNA bp)* | **Protein Percentages** | |
|  |  |  |  | **Identity** | **Similarity** |
| PPE5  PPE6 | Frameshift | Frameshift | Frameshift | 100%  99.9% | 100%  99.9% |
| PPE7  PPE8 | Frameshift | Frameshift | Frameshift | 98.6%  99.9% | 99.3%  99.9% |
| PPE10 | - | 1 (40, E-K) | - | 99.8% | 100% |
| PPE12 | - | - | - | 100% | 100% |
| PPE13 | - | - | - | 100% | 100% |
| PPE16 | 1 (87, V-A) | - | - | 99.8% | 99.8% |
| PPE21 | 1 (258, G-D) | - | - | 99.9% | 99.9% |
| PPE24 | - | - | 1 (488, 156 bp) | 99.9% | 99.9% |
| PPE28 | 1 (150, V-A) | - | - | 99.8% | 99.8% |
| PPE34 | - | 2 (653, A-S, 1290, D-N) | 3 (615, 75 bp, 1126, 138 bp, 1298, 120 bp) | 99.6% | 99.8% |
| PPE35 | - | - | - | 100% | 100% |
| PPE39 | - | - | - | 100% | 100% |
| PPE40 | - | - | - | 100% | 100% |
| PPE42 | 1 (53, T-A) | - | - | 99.8% | 99.8% |
| PPE52 | 1 (226, G-S) | - | - | 99.8% | 99.8% |
| PPE53 | 1 (33, G-R) | - | - | 99.8% | 99.8% |
| PPE70** | - | - | - | - | - |
| PPE54 | 1 (103, A-E) frameshift | 1 (375, Q-E) frameshift | 1 (422, 5283 bp) | - | - |
| PPE55 | - | - | - | - | - |
| PPE56 | - | - | - | - | - |
| PPE62 | - | - | - | 100% | 100% |
| PPE64 | - | - | - | 99.5% | 99.6% |

* Change from CDC1551 to H37Rv

** PPE70 is not present in *M. tuberculosis* H37Rv and was identified after the initial annotation of H37Rv in CDC1551. This gene is situated between PPE53 and PPE54.
